# *SOS1* Mutations in Noonan Syndrome: Molecular Spectrum, Structural Insights on Pathogenic Effects, and Genotype–Phenotype Correlations

**DOI:** 10.1002/humu.21492

**Published:** 2011-03-08

**Authors:** Francesca Lepri, Alessandro De Luca, Lorenzo Stella, Cesare Rossi, Giuseppina Baldassarre, Francesca Pantaleoni, Viviana Cordeddu, Bradley J Williams, Maria L Dentici, Viviana Caputo, Serenella Venanzi, Michela Bonaguro, Ines Kavamura, Maria F Faienza, Alba Pilotta, Franco Stanzial, Francesca Faravelli, Orazio Gabrielli, Bruno Marino, Giovanni Neri, Margherita Cirillo Silengo, Giovanni B Ferrero, Isabella Torrrente, Angelo Selicorni, Laura Mazzanti, Maria C Digilio, Giuseppe Zampino, Bruno Dallapiccola, Bruce D Gelb, Marco Tartaglia

**Affiliations:** 1 IRCCS Casa Sollievo della SofferenzaLaboratorio Mendel, San Giovanni Rotondo, Italy; 2 Dipartimento di Scienze e Tecnologie Chimiche, Università “Tor Vergata,”Rome, Italy; 3 UO Genetica Medica, Policlinico S.Orsola-MalpighiBologna, Italy; 4 Dipartimento di Pediatria, Università di TorinoTurin, Italy; 5 Dipartimento di Ematologia, Oncologia e Medicina Molecolare, Istituto Superiore di SanitàRome, Italy; 6 GeneDxGaithersburg, Maryland; 7 Medical Genetics, Federal University of Sao PauloSao Paulo, Brazil; 8 Department of Biomedicine of Developmental Age, University of BariBari, Italy; 9 AuxoendocrinologiaOspedale Pediatrico, Brescia, Italy; 10 Servizio aziendale di Consulenza GeneticaOspedale di Bolzano, Italy; 11 S.C. Genetica UmanaOspedali Galliera, Genova, Italy; 12 Istituto di Scienze Materno-Infantili, Università Politecnica delle MarcheAncona, Italy; 13 Division of Pediatric Cardiology, Department of Pediatrics, “Sapienza” UniversityRome, Italy; 14 Istituto di Genetica Medica, Università Cattolica del Sacro CuoreRome, Italy; 15 Clinica Pediatrica, Università Milano Bicocca A.O. S Gerardo Fondazione MBBMMonza, Italy; 16 Dipartimento di Pediatria, Università degli Studi di BolognaBologna, Italy; 17 Ospedale Pediatrico “Bambino Gesù,”IRCCS, Rome, Italy; 18 Istituto di Clinica Pediatrica, Università Cattolica del Sacro CuoreRome, Italy; 19 Child Health and Development Institute, Mount Sinai School of MedicineNew York, New York

**Keywords:** Noonan syndrome, NS, *SOS1*, mutation analysis, structural analysis, genotype–phenotype correlations

## Abstract

Noonan syndrome (NS) is among the most common nonchromosomal disorders affecting development and growth. NS is caused by aberrant RAS-MAPK signaling and is genetically heterogeneous, which explains, in part, the marked clinical variability documented for this Mendelian trait. Recently, we and others identified *SOS1* as a major gene underlying NS. Here, we explored further the spectrum of *SOS1* mutations and their associated phenotypic features. Mutation scanning of the entire *SOS1* coding sequence allowed the identification of 33 different variants deemed to be of pathological significance, including 16 novel missense changes and in-frame indels. Various mutation clusters destabilizing or altering orientation of regions of the protein predicted to contribute structurally to the maintenance of autoinhibition were identified. Two previously unappreciated clusters predicted to enhance SOS1's recruitment to the plasma membrane, thus promoting a spatial reorientation of domains contributing to inhibition, were also recognized. Genotype–phenotype analysis confirmed our previous observations, establishing a high frequency of ectodermal anomalies and a low prevalence of cognitive impairment and reduced growth. Finally, mutation analysis performed on cohorts of individuals with nonsyndromic pulmonic stenosis, atrial septal defects, and ventricular septal defects excluded a major contribution of germline *SOS1* lesions to the isolated occurrence of these cardiac anomalies. Hum Mutat 32:760–772, 2011. © 2011 Wiley-Liss, Inc.

## Introduction

Noonan syndrome (NS; MIM♯ 163950) is a relatively common and clinically variable disorder characterized by postnatal reduced growth, facial dysmorphism, and congenital heart defects (CHDs) [Allanson, 1987; Noonan, 1994; Tartaglia et al., 2010; van der Burgt, 2007]. The distinctive and most recurrent facial features consist of a broad forehead, hypertelorism, down-slanting palpebral fissures, ptosis, high arched palate, and low-set, posteriorly rotated ears. Cardiac involvement is present in up to 80–90% of affected individuals, with pulmonic stenosis (PS), septal defects, and hypertrophic cardiomyopathy (HCM) occurring most commonly [Burch et al., 1993; Marino et al., 1999]. Other associated features include multiple skeletal defects (chest and spine deformities), webbed/short neck, variable cognitive deficits, cryptorchidism, lymphatic dysplasia, bleeding diathesis, and, rarely, predisposition to certain hematologic malignancies during childhood.

NS is genetically heterogeneous, and, based upon the recent discoveries of the underlying disease genes, is now regarded as a disorder caused by enhanced signal flow through the RAS-MAPK pathway [Tartaglia et al., 2010]. This signaling cascade is known to mediate diverse biological functions, including cell proliferation, survival, fate determination, and differentiation. It is activated in response to cytokine, hormone, and growth factor stimulation, and is a major mediator of early and late developmental processes including morphology determination, organogenesis, synaptic plasticity processes, and growth. In approximately 50% of affected individuals, NS is caused by heterozygous missense mutations in the *PTPN11* gene [Tartaglia et al. 2001, 2002], which encodes a cytoplasmic protein tyrosine phosphatase positively modulating RAS function. Activating mutations in five additional genes coding for transducers or modulatory proteins participating in this signaling pathway (i.e., *KRAS*, *NRAS*, *SOS1*, *RAF1*, and *BRAF*) account for an additional one-fourth of NS cases [Tartaglia et al., 2010]. Mutations in these and other functionally related genes (i.e., *CBL*, *NF1*, *KRAS*, *HRAS*, *BRAF*, *SPRED1*, *SHOC2*, *MAP2K1*, and *MAP2K2*) have been reported to underlie clinically related disorders [Aoki et al., 2008; Tartaglia et al., 2011; Tidyman and Rauen, 2009]. Genotype–phenotype correlation surveys have documented that the substantial phenotypic variation characterizing NS can be ascribed, in part, to the gene mutated and even to the specific molecular lesion involved.

**Figure 1:**
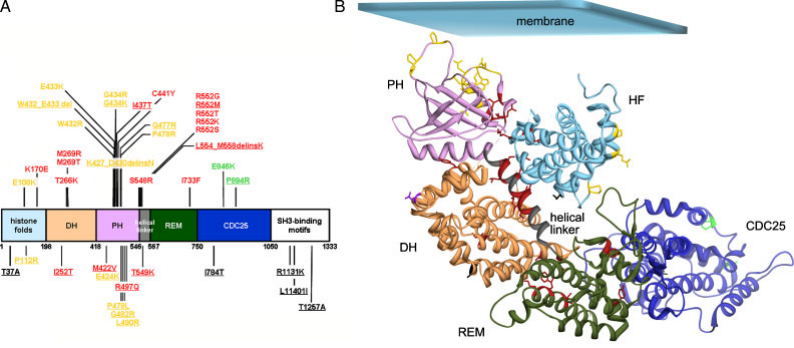
SOS1 domain structure and location of residues altered in Noonan syndrome. **A:** Schematic structure of SOS1 and variants identified in the present study. SOS1 protein domains are indicated (DH, DBL homology domain; PH, pleckstrin homology domain; REM, RAS exchanger motif; CDC25, CDC25 domain). Disease-causing mutations and probably pathogenetic/unclassified variants are shown above and below the cartoon, respectively. Residues affected by class 1 mutations/variants are shown in red, while those affected by class 2 and class 3 changes are shown in yellow and green, respectively. Residues affected by substitutions with unpredictable effect on SOS1 function are shown in black. Novel amino acid substitutions are underlined. **B:** Location of affected residues in SOS1 represented in its inactive conformation, according to the crystal structure of the protein truncated at the C-terminus (residues 1–1049) (PDB ID: 3KSY) [Guerasko et al., 2010]. Cα ribbon trace of the HF (sky blue), DH (sandy brown), PH (plum), REM (dark green), and CDC25 (blue) domains, and the helical linker connecting the PH and REM domains (gray). Mutated residues are indicated with their side chains as thick lines and colored as reported above. Residue Asp309 (uncharacterized mutation p.Asp309Tyr) is shown in purple. Affected residues are listed in Supp. Table S2.

We and others recently reported that missense mutations in *SOS1* (MIM♯ 182530) account for a significant proportion of NS [Roberts et al., 2007; Tartaglia et al., 2007; Zenker et al., 2007a]. *SOS1* encodes a guanine nucleotide exchange factor (GEF) responsible for stimulating the conversion of RAS from the inactive, GDP-bound to the active, GTP-bound form [Nimnual and Bar-Sagi, 2002]. SOS1 is a large multidomain protein characterized by an N-terminal regulatory portion including tandem histone-like folds (HF), which are followed by a Dbl-homology (DH) domain and a pleckstrin-homology (PH) domain, and a C-terminal catalytic region including the RAS exchanger motif (REM) and CDC25 domains, followed by a tail providing docking sites for adaptor proteins required for receptor anchoring (Fig. 1). The majority of NS-causing *SOS1* mutations were observed to affect residues predicted to be implicated in the maintenance of SOS1 in its autoinhibited conformation, and the first biochemical characterizations of mutants consistently documented enhanced protein function and increased signal flow through RAS [Roberts et al., 2007; Tartaglia et al., 2007]. These surveys also indicated that subjects heterozygous for a mutated *SOS1* allele tend to exhibit a distinctive phenotype that is characterized by ectodermal abnormalities generally associated with an absence of cognitive deficits [Tartaglia et al., 2007; Zenker et al., 2007a]. We also observed that height was less frequently below the third centile compared with the overall NS population [Tartaglia et al., 2007]. Although available information supports the view that *SOS1* is not mutated in cardiofaciocutaneous syndrome (CFCS; MIM♯ 115150) [Zenker et al., 2007a], a condition clinically related to NS, a few individuals with ectodermal manifestations and distinctive facial dysmorphism that might be suggestive of CFCS have recently been reported has having *SOS1* mutations [Narumi et al., 2008; Nystrom et al., 2008]. In these subjects, cognitive deficits were generally absent or minor, but at least one case with mental retardation was reported [Narumi et al., 2008].

Here, we characterized further the molecular spectrum and distribution of *SOS1* mutations, as well as the phenotypic features associated with those molecular defects. We also explored possible involvement of germline *SOS1* mutations in an opportunely selected group of isolated CHDs that occur as an associated feature in subjects with NS and heterozygous for a *SOS1* mutation. Our results provide a more accurate description of the spectrum of *SOS1* gene defects, their consequence on SOS1 structure and function, and their associated clinical features.

## Materials and Methods

### Patients

Four cohorts were included in the study. The first cohort (group 1) included 143 clinically well-characterized patients with NS enrolled in research protocols. Nearly all subjects of this cohort were of European ancestry, with the majority being Italian. Within this group, subjects were assessed by clinical geneticists experienced with NS and clinically related disorders (G.Z., M.C.D., L.M., B.D., G.B.F., M.C.S., A.S., I.K., G.N., M.F.F., A.P., F.S. and O.G.). Clinical assessment included physical, anthropometric, neurologic, and cardiac evaluations, as well as accurate examination for craniofacial features, ophthalmologic, and otorhinolaryngologic defects, and ectodermal and musculoskeletal anomalies. Clinical features for the majority of these individuals satisfied diagnostic criteria reported for NS [van der Burgt et al. 1994], but a few individuals with a highly suggestive phenotype who lacked sufficient features to receive a definitive diagnosis were also included. Based on scanning of the coding exons by denaturing high-performance liquid chromatography (DHPLC) analysis and/or direct sequencing, no subject within this cohort harbored a mutation in *PTPN11*, *KRAS*, or *RAF1*. For approximately half of the cases, mutations in *BRAF*, *MAP2K1*, *SHOC2*, *CBL,* and *NRAS* had also been excluded. Besides this large cohort, nine subjects with features fitting CFCS and no mutation in *KRAS*, *BRAF*, *MAP2K1,* or *MAP2K2* (group 2) [Sarkozy et al., 2009a] were also included in the study. The third cohort (group 3) (*N* = 358) comprised anonymous samples from individuals with phenotypes suggestive of NS for whom commercial DNA diagnostic testing was performed. Clinical data were not available for these patients, and *PTPN11* mutations had not systematically been excluded in all the subjects included in this group. Although the output obtained from genotyping this cohort of subjects could not be used in genotype–phenotype correlation studies or to estimate *SOS1* mutation prevalence in the NS population, the mutation data were utilized to provide a more detailed picture about the molecular spectrum of disease-causing mutations affecting the *SOS1* gene. Finally, a cohort of 59 subjects with nonsyndromic CHDs (PS, *N* = 21; atrial septal defects (ASDs), *N* = 23; ventricular septal defects (VSDs), *N* = 15) was included in the study (group 4). Clinical assessment of patients with isolated CHDs included complete physical evaluation of dysmorphism and malformations, anthropometric measurements, renal ultrasonography, and radiological studies. Cardiac evaluation included preoperative physical evaluation, chest X-ray film, 12-lead electrocardiogram, and two-dimensional transthoracic echocardiography with color flow Doppler. Karyotype analysis was performed in all patients of this cohort. Inclusion criteria were based on the absence of any association with other clinical features, and chromosomal anomalies, including the 22q11 deletion.

Informed consent for the genetic analyses was obtained from all patients or their legal guardians.

### Mutation Analysis

Genomic DNA was isolated from peripheral blood leukocytes according to standard procedures. The entire *SOS1* coding sequence, as well as the exon/intron boundaries and flanking intronic portions were scanned for mutations by direct sequencing (group 3) or DHPLC analysis (groups 1, 2, and 4) with the use of the 3100 and/or 3500HT Wave DNA Fragment Analysis System (Transgenomic, Omaha, NE), at column temperatures recommended by the Navigator version 1.5.4.23 software (Transgenomics), as previously described [Tartaglia et al., 2007]. Amplimers having abnormal elution profiles were reamplified, purified (Qiagen, Hilden, Germany) and sequenced using the ABI BigDye Terminator Sequencing Kit v.1.1 (Applied Biosystems, Foster City, CA) and an ABI 3700 Capillary Array Sequencer or ABI 3100 Genetic Analyzer (Applied Biosystems). Primer pair sequences as well as PCR and DHPLC analysis settings are available upon request. Length of deletions and dinucleotide mutations were determined by cloning purified PCR products in a pCR 2.1 TOPO vector (Invitrogen, Carlsbad, CA) and sequencing purified clones (Plasmid Mini Kit, Qiagen). Nucleotide numbering of the mutations and exonic disease-unrelated variants reflects cDNA numbering with 1 corresponding to the A of the ATG translation initiation codon in the reference sequence (NM_005633.3), whereas position of the intronic variants were numbered according to the reference genomic sequence (NG_007530.1).

The level of conservation of affected residues among orthologous *SOS1* genes was evaluated by using the NCBI HomoloGene tool (http://www.ncbi.nlm.nih.gov/homologene), while the SIFT (Sorting Intolerant From Tolerant, http://blocks.fhcrc.org/sift/SIFT.html) [Ng and Henikoff, 2001] and PolyPhen (Polymorphism Phenotyping, http://genetics.bwh.harvard.edu/pph/) [Sunyaev et al., 2000] software programs were used to predict the biological relevance of the identified missense variants on protein function.

When available, parental DNAs were sequenced to establish whether the identified changes in sporadic cases were de novo or to confirm cosegregation between the variant and phenotype in families transmitting the trait. Paternity was confirmed using the AmpDESTER ProfilerPlus kit (Applied Biosystems). All missense changes proven to be de novo by parental DNA genotyping were deemed mutations causally linked to the disorder. To exclude the possibility that variants cosegregating with NS were neutral polymorphic changes occurring in the population, at least 300 population-matched DNAs obtained from unaffected subjects were screened (DHPLC analysis and sequencing of variant elution profiles). When parental DNAs were not available, we considered a novel nonsynonymous variant as a causative mutation when it affected a residue already reported to be mutated. Novel variants involving residues localized in close proximity to residues mutated in NS, predicted to affect protein structure/function by Polyphen, SIFT, and structural evidence, but without sufficient genetic evidence (i.e*.*, cases with unreported ethnicity or missing clinical evaluation and/or genetic testing of other members of their families) were considered as probably pathogenic. These missense changes were found not to occur in controls, and resulted in a nonconservative substitution at an invariant or highly conserved residue (*Homo sapiens* [NP_005624.2], *Pan troglodytes* [XP_515425.2], *Canis familiaris* [XP_540157.2], *Bos taurus* [XP_617859.4], *Mus musculus* [NP_033257.2], *Rattus norvegicus* [XP_233820.4], and *Drosophila melanogaster* [NP_476597.2]). Finally, a third category grouping functionally unclassified variants was considered for those novel changes for which structural analysis did not allow to infer any functional effect.

The position of mutated amino acid residues was modeled by using the recently generated crystal structure of the SOS1 protein truncated at the *C*-terminus (residues 1–1049) deposited in the RCSB Protein Data Bank (http://www.rcsb.org/pdb/home/home.do) (PDB ID: 3KSY) [Guerasko et al., 2010]. Figures were prepared using the UCSF Chimera 1.5 software package (http://www.cgl.ucsf.edu/chimera/) [Pettersen et al., 2004]. Electrostatic potential calculations were performed with the APBS software [Baker et al., 2001].

### Statistical Analyses

Confidence intervals for proportions were calculated by means of VassarStats software (http://faculty.vassar.edu/lowry/VassarStats.html) using the Newcombe-Wilson method including continuity correction [Newcombe, 1998; Wilson, 1927]. Genotype–phenotype correlations were performed using 2 × 2 contingency table analysis. The significance threshold was set at *P* = 0.05.

## Results

### *SOS1* Mutation Scanning in NS, CFCS, and Isolated CHD

DHPLC analysis and bidirectional direct sequencing of the entire *SOS1* coding sequence on peripheral blood leukocyte genomic DNA specimens of groups 1 and 3 identified 41 different heterozygous missense nucleotide substitutions and three small in-frame indels in 108 unrelated subjects. Among them, five were private or common variants unrelated with the trait. Three of them, c.1964C>T (p.Pro655Leu), c.3032A>G (p.Asn1011Ser), and c.3959A>G (p.His1320Arg, had previously been reported in unaffected individuals [Roberts et al., 2007; Tartaglia et al., 2007]. Similarly, the c.1705C>G (p.Leu569Val) was documented to occur as a homozygous change in an unaffected parent, and the c.2122G>A (p.Ala708Thr) was found to occur as a polymorphic change in ethnic-matched unaffected individuals of Hispanic ancestry, and were therefore regarded as benign polymorphisms. A full list of the disease-unrelated changes, including silent nucleotide substitutions and intronic variants, is reported in Supp. Table S1.

In these two cohorts, 25 *SOS1* sequence variants were deemed to be of pathological significance (Table 1). Among these, 17 had previously been established as causative mutations (Supp. Table S2), whereas 8 were novel. Among the latter, five mutations were single nucleotide substitutions, but three small in-frame indels affecting two mutational hot spots within the PH domain (p.Lys427_Asp430delinsAsn and p.Trp432_Glu433del) and the helical linker connecting the PH and REM domains (PH-REM linker) (p.Leu554_Met558delinsLys) were also identified. The c.1300_1301GG>AA (p.Gly434Lys) and c.1310T>C (p.Ile437Thr) changes were demonstrated to occur as de novo events by genotyping of parental DNAs. The c.2681C>G transition (p.Pro894Arg) was found to cosegregate with the disease, whereas the remaining two variants, c.1430A>G (p.Gln477Arg) and c.1655G>T (p.Arg552Met), affected amino acid residues that had previously been reported to be mutated in NS (Supp. Table S2). Eight novel missense changes (p.Pro112Arg, p.Ile252Thr, p.Met422Val, p.Glu424Lys, p.Gly482Arg, p.Leu490Arg, p.Arg497Gln, and p.Thr549Lys) were considered as probably pathogenic mutations. The majority of these variants were not observed in more than 300 population-matched unaffected individuals, and were nonconservative, the majority affecting invariant residues among SOS1 orthologs and predicted to be “damaging” or have functional relevance according to the PolyPhen or/and SIFT programs. Moreover, affected residues were located in mutational hot spots or in close spatial proximity to residues mutated in NS and were predicted to have structural/functional consequences on protein structure (see below). For these variants, however, the evidence provided by the theoretical models and structural analysis was not unambiguously supported by genetic evidence. Finally, six missense nucleotide substitutions (p.Thr37Ala, p.Pro478Leu, p.Ile784Thr, p.Arg1131Lys, p.Leu1140Ile, and p.Thr1257Ala) were classified as variants of unknown significance. For those, parental DNAs were not submitted for testing or no clinical information was available for the carrier parent, and the predicted amino acid change was generally not considered as having functional relevance according to the PolyPhen or/and SIFT programs. Most of these changes affected the C-terminal portion of the protein for which no structural information is currently available, precluding any consideration regarding possible consequences on protein function.

**Table 1:** *SOS1* Exonic Indels and Missense Changes Identified in the Study

Exon	Nucleotide change	Amino acid change	Domain	Notes	Number of cases
*Mutations*					
4	c.322G>A	p.Glu108Lys	HF		2, fam.unknown
5	c.508A>G	p.Lys170Glu	HF		1, sporadic; 2, fam.unknown
7	c.797C>A	p.Thr266Lys	DH		1, sporadic; 3, fam.unknown
7	c.806T>C	p.Met269Thr	DH		3, sporadic; 2, fam.unknown
7	c.806T>G	p.Met269Arg	DH		1, fam.unknown
11	**c.1281_1289delGAATATTGA**	**p.Lys427_Asp430delinsAsn**	PH		1, fam.unknown
11	c.1294T>C	p.Trp432Arg	PH		1, sporadic; 1, fam.unknown
11	**c.1294_1299delTGGGAG**	**p.Trp432_Glu433del**	PH	CTRL, NPS	1, sporadic
11	c.1297G>A	p.Glu433Lys	PH		3, sporadic; 3, fam.unknown
11	c.1300G>A	p.Gly434Arg	PH		2, fam.unknown
11	**c.1300_1301delGGinsAA**	**p.Gly434Lys**	PH	de novo, PPhen+, SIFT+, CON	1, sporadic
11	**c.1310T>C**	**p.Ile437Thr**	PH	de novo, PPhen++, SIFT+, CON	1, sporadic; 1, familial
11	c.1322G>A	p.Cys441Tyr	PH		1, fam.unknown
11	**c.1430A>G**	**p.Gln477Arg**	PH	PPhen−, SIFT−, CON	2, fam.unknown
11	c.1433C>G	p.Pro478Arg	PH		1, sporadic
11	c.1642A>C	p.Ser548Arg	PH-REM linker		4, fam.unknown
11	c.1654A>G	p.Arg552Gly	PH-REM linker		3, sporadic; 8, fam.unknown
11	c.1655G>A	p.Arg552Lys	PH-REM linker		2, sporadic; 2, fam.unknown
11	**c.1655G>T**	**p.Arg552Met**	PH-REM linker	NPS, PPhen++, SIFT+, CON	2, sporadic
11	c.1655G>C	p.Arg552Thr	PH-REM linker		1, fam.unknown
11	c.1656G>C	p.Arg552Ser	PH-REM linker		2, sporadic; 7, fam.unknwn
11	**c.1660_1673delCTTGATGTAACAATinsAA**	**p.Leu554_Met558delinsLys**	PH-REM linker		1, fam.unknown
15	c.2197A>T	p.Ile733Phe	REM		1, fam.unknown
17	c.2536G>A	p.Glu846Lys	CDC25		1, sporadic, 6 fam.unknown
18	**c.2681C>G**	**p.Pro894Arg**	CDC25	CTRL, PPhen+, SIFT−, CON	1, familial^a^
*Possibly pathogenic variants*					
4	**c.335C>G**	**p.Pro112Arg**	HF	NPS, PPhen+, SIFT−	1, sporadic
7	**c.755T>C**	**p.Ile252Thr**	DH	CTRL, PPhen++, SIFT+, CON	1, familial
11	**c.1264A>G**	**p.Met422Val**	PH	PPhen++, SIFT−; CON	1, fam.unknown
11	**c.1270G>A**	**p.Glu424Lys**	PH	PPhen+, SIFT+, CON	1, fam.unknown
11	**c.1444G>C**	**p.Gly482Arg**	PH	PPhen+, SIFT+, CON	1, fam.unknown
11	**c.1469T>G**	**p.Leu490Arg**	PH	CTRL, NPS, PPhen++, SIFT+, CON	1, sporadic
11	**c.1490G>A**	**p.Arg497Gln**	PH	CTRL, PPhen+, SIFT+, CON	1, sporadic^b^
11	**c.1646C>A**	**p.Thr549Lys**	PH-REM linker	PPhen+, SIFT−, CON	1, fam.unknown
*Unclassified variants*					
3	**c.109A>G**	**p.Thr37Ala**	HF	PPhen−, SIFT−, CON	1, fam.unknown
11	c.1433C>T	p.Pro478Leu	PH	PPhen+, SIFT-, CON	1, fam.unknown
15	**c.2351T>C**	**p.Ile784Thr**	REM	PPhen++, SIFT+, CON	1, fam.unknown^c^
23	**c.3392G>A**	**p.Arg1131Lys**	*C*-terminus	PPhen−, SIFT−, CON	1, fam.unknown
23	**c.3418T>A**	**p.Leu1140Ile**	*C*-terminus	PPhen−, SIFT−, CON	1, fam.unknown
24	**c.3769A>G**	**p.Thr1257Ala**	*C*-terminus	PPhen−, SIFT−, CON	1, fam.unknown

Nucleotide numbering reflects cDNA numbering with 1 corresponding to the A of the ATG translation initiation codon in the reference sequence (NM_005633.3). Exon 2 corresponds to the first protein coding exon. Novel mutations are in bold.

a Variant inherited from an affected parent.

b Variant inherited from an apparently unaffected parent.

c Variant concomitant with the disease-causing p.Met269Arg change.

HF, histone folds; DH, DBL homology domain; PH, plekstrin homology domain; REM, RAS exchanger motif, CDC25, CDC25 domain. Fam.unknown, familial status unknown; NPS, unavailable parental DNA samples. CTRL, variant not occurring in ≥300 population-matched unaffected subjects; de novo, variant demonstrated to occur de novo by DNA genotyping of unaffected parents; PPhen++, amino acid change predicted to be “probably damaging” by PolyPhen; PPhen+, amino acid change predicted to be “possibly damaging” by PolyPhen; PPhen−, amino acid change predicted to be “benign” by Polyphen; SIFT+, amino acid change predicted to “affect protein function” by SIFT; SIFT−, amino acid change predicted to be “tolerated” by SIFT; CON, variant at a conserved residue.

*SOS1* mutations were identified in 26 of the 143 subjects (18.2%) of group 1, which included patients with clinical features satisfying the diagnostic criteria of NS or highly suggestive for the disorder, and negative for mutations in *PTPN11*, *RAF1*, and *KRAS*. Based on the accurate clinical assessment of this cohort and the relative prevalence of mutations affecting those disease genes in NS, this finding supports the view that mutations in the *SOS1* gene account for approximately 10% of NS cases, which is in line with our previous estimate [Tartaglia et al., 2007], but slightly below those obtained from two other clinically well characterized NS cohorts [Roberts et al., 2007; Zenker et al., 2007a].

DHPLC mutation scanning of coding exons, splice junctions, and flanking intronic portions of *SOS1* did not reveal any putative pathogenic mutation in the nine subjects with a diagnosis of CFCS and negative for mutations in *BRAF*, *KRAS*, *MAP2K1*, and *MAP2K2*. Similarly, no mutation was identified among the 59 patients with nonsyndromic CHDs included in the study, excluding a major involvement of germline *SOS1* mutations in PS (*N* = 21; 95% confidence interval [CI] = 0.000–0.192), ASD (*N* = 23, 95% CI = 0.000–0.178), and VSD (*N* = 15, 95% CI = 0.000–0.253).

### *SOS1* Mutation Diversity, Cluster Distribution, and Molecular Modeling Analysis

The present data and available published records (updated to July 2010) (Supp. Table S2) were utilized to analyze the diversity of NS-causing mutations, their cluster distribution, and to explore their effects on SOS1 structure and function. The 183 reported missense mutations (including those with probably damaging effects) were observed to affect 32 residues, which were not randomly scattered along the protein but tended to cluster in specific regions (Fig. 1). Approximately 40% of *SOS1* defects affected four residues located in the PH-REM linker (Ser548, Thr549, Leu550, and Arg552), with substitutions of residue Arg552 accounting for one-third of all mutations. Another mutation cluster, involving short stretches within the PH domain (i.e., Glu424, Trp432, Glu433, and Gly434, and Gln477, Pro478, and Gly482) explained roughly 20% of mutations. A third functional cluster (see below) resided at the interacting regions of the DH (Thr266 and Met269) and REM (Lys728, Trp729, and Ile733) domains (16% of total mutations). Among the most recurrent mutations, the c.2536G>A transition, predicting the substitution of Glu846 by lysine within the CDC25 domain, accounted for 10% of defects. Such a skewed distribution of affected residues implies specific perturbing consequences on protein function.

SOS1's GEF activity is controlled principally by two binding sites for RAS: the catalytic site, which is located entirely within the CDC25 domain and promotes GDP release from RAS, and a distal site, which is bracketed by the CDC25 domain and REM domains and positively modulates GEF activity through promotion of a conformational change at the active site that allows substrate GDP-RAS to bind [Margarit et al., 2003]. Basally, the catalytic output of SOS1 is constrained by the N-terminal regulatory HF domain and DH-PH unit [Guerasko et al., 2010; Sondermann et al., 2004], and structural data indicate that this autoinhibitory effect is exerted through direct HF and DH domain-mediated blockade of the allosteric site. An extensive interdomain binding network also involving the PH-REM helical linker and the PH domain has been recognized to stabilize this inhibitory conformation. Following the translocation of SOS1 to the membrane, the inhibitory effect of the HF and DH domains is relieved allowing RAS binding to the allosteric site, which in turn, promotes a conformational rearrangement of the CDC25 domain allowing RAS binding to the catalytic site. Based on this allosteric mechanism of activation and recent evidence indicating that the HF domain and the DH-PH unit are conformationally coupled to control SOS1's recruitment to the plasma membrane and release of autoinhibition [Guerasko et al., 2010], the distribution of identified mutations in NS supports a picture in which the vast majority of disease-causing lesions affect the stability of the intramolecular interactions that maintain SOS1 in an autoinhibited state by at least two major distinct mechanisms.

**Figure 2:**
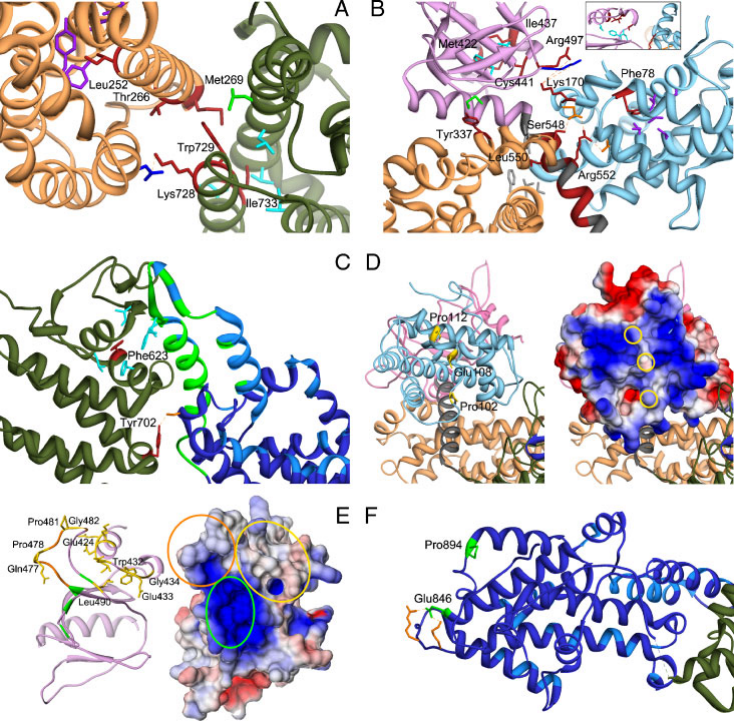
Detailed analysis of structural perturbations resulting from Noonan syndrome-causing amino acid substitutions. **A:** Class 1 mutations affecting residues at the distal RAS binding site. The cartoon includes the DH (sandy brown) and REM (dark green) domains only. Affected residues are shown in red. The autoinhibitory binding network includes the hydrophobic interaction between residues Met269 (DH) and Trp729 (REM), both mutated in NS, and between the former and Leu687 (green). Leu690, Val697, Ile718, and Ile736 are hydrophobic residues (cyan) that interact with Ile733 (REM). Mutated residues Thr266 (DH) and Lys728 (REM) face each other. Substitution of Thr266 by lysine would create an electrostatic repulsion with Lys728. The unclassified variant Leu252 contributes to a hydrophobic core with residues Tyr215, Leu219, Ile249, Tyr295, and Tyr298 (purple), whose disruption is expected to perturb considerably the DH domain surface interacting with the REM domain. **B:** Class 1B mutations. The cartoon includes the HF (sky blue), DH (sandy brown), and PH (magenta) domains and the PH-REM helical linker (gray). Relevant affected residues are shown in red. Met422 and Ile437 (PH) participate in a hydrophobic bonding network with residues Ile425, Phe464, and Leu467 (cyan, see also the inset). Hydrophobic interaction between Tyr337 and Met538 (green) contributes to the binding network stabilizing the interaction between the PH and DH domains. Other interdomain interactions involve Leu550 and residues of the DH and PH domains, Val225, Leu221, Phe226, and Tyr546 (gray), Arg552, and residues of the HF domain, Asp140 and Asp169 (orange), Ser548 and Asp169, and Lys170, and residues of the PH domain, Arg497 and Lys498 (blue). Phe78 participates to the hydrophobic interaction involving residues of the HF domain core located close to the PH domain and PH-REM linker (Leu55, Leu59, Val74, Val133, and Ile137; purple). **C:** Class 1 mutations affecting the REM domain region (dark green) interacting with the CDC25 domain (blue). The helical hairpin (residues 929–978) implicated in the conformational switch (green) and residues interacting with RAS at the active site (light blue) are shown. Phe623 hydrophobically interact with Ile 601, Leu613, Phe627, Ile956, and Phe958 (cyan). The hydrogen bond between Tyr702 and Ser802 (orange) is also shown. **D:** Class 2 mutations affecting the HF domain. The left panel shows the HF (light blue), DH (sandy brown), and PH (plum) domains. The HF surface colored according to the electrostatic potential (from red at −3kT/e to blue at +3kT/e) is also shown (right panel). Mutations affect solvent exposed residues (yellow side chains, left panel; yellow circles, right panel) located in a region that has a positive electrostatic potential (right panel), and has been implicated in membrane binding. **E:** Class 2 mutations affecting the PH domain. The left panel includes the PH domain (plum) only. The PH surface, colored according to the electrostatic potential (from red at −5kT/e to blue at +5kT/e) is also shown (left panel). Affected residues are shown in yellow (side chains, left panel; circles, right panel). Residues that are predicted to bind to PIP_2_ [Zheng et al., 1997], Lys456, Arg459, Lys472, and Arg489, are shown in green (circled in the right panel), whereas the PA-interacting region (residues 472–483) [Zhao et al., 2007] is shown in orange (circled in the right panel). **F:** Class 3 mutations. The cartoon includes the REM (dark green) and CDC25 (blue) domains only (residues 567–1049). Affected residues are shown in green. Residues implicated in RAS binding at the catalytic site are shown (light blue). Glu846 and Pro894 are placed distally from the active site and regions implicated in the conformational rearrangement of the CDC25 domain. Glu846 electrostatically interacts with Arg1026 and Lys 1029 (orange).

A first class of mutations includes lesions predicted to promote conformational rearrangements of domains that reduce the enzyme self-inhibition by impairing proper masking of the distal RAS binding site or by acting on the allosteric control of catalytic activity. Within this class, a first group of mutations (Class 1A: p.Thr266Lys, p.Met269Arg/Thr, p.Lys728Ile, p.Trp729Leu, and p.Ile733Phe) involve residues that participate in the autoinhibitory interaction of the DH and REM domains blocking RAS access at the allosteric site, or are closely located to them (Fig. 2A). These mutations are predicted to affect the stability of the inactive conformation of the protein directly by disrupting the inhibitory interdomain bonding network at the distal site. Among these, the most recurrent mutations affect Met269 (10% of total cases), which interacts directly with residues of the REM domain implicated in RAS binding [Sondermann et al., 2004]. Of note, Lys728 and Trp729 are among these, and Ile733 is very close to these residues; therefore, their substitution could also affect the REM's affinity for GTP-bound RAS in addition to the stability of the DH-REM interface. Indeed, the drastic p.Trp729Glu substitution has been shown to inhibit RAS binding to the REM domain [Sondermann et al., 2004]. This might be related to the fact that within this group, mutations affecting residues located at the DH surface appear to be more common (15% of total changes) compared to those affecting the REM surface (2%). Among the probably pathogenetic variants, the p.Ile252Thr substitution could be included in this group. Ile252 is an invariant residue placed within a hydrophobic core of the DH domain, which also involves Thr215, Leu219, Ile249, Tyr295, and Tyr298. Structural perturbation of the DH fold is expected to destabilize the masking of the distal RAS binding site.

A second mutation group includes lesions affecting the interaction between the HF, DH, and PH domains, thus perturbing the overall autoinhibited conformation in which the HF and DH domains block the distal RAS binding site [Guerasko et al., 2010] (Class 1B: p.Lys170Glu, p.Tyr337Cys, p.Ile437Thr, p.Cys441Tyr, p.Ser548Arg, p.Leu550Pro, p.Arg 552Gly/Thr/Met/Lys/Ser, p.Leu554_Met558delinsLys, and probably pathogenetic variants p.Met422Val, p.Arg497Gln, and p.Thr549Lys) (Fig. 2B). Within this group, mutations of residues 548–558 are the most recurrent. This stretch corresponds to the helical linker connecting the PH and REM domains, which contributes structurally to the maintenance of the autoinhibited conformation by interacting with the HF and DH domains [Guerasko et al., 2010]. For instance, Arg552 interacts directly with the side chains of Asp140 and Asp169 of the HF domain, and mutation of either Arg552 or Asp140 was demonstrated to completely abolish the interaction between these domains [Sondermann et al., 2005]. A similar effect might be predicted for the recurrent Lys-to-Glu change affecting codon 170 within the HF domain, and possibly for the p.Arg497Gln substitution in the PH domain, because the electrostatic interactions between these two residues located at the HF-PH domain interface (but also close to the helical linker) contribute to stabilizing the HF domain's orientation. Similarly, substitution of residues Ile437 and Cys441 might affect the PH domain's structure in the region of the domain facing toward the HF domain and possibly cause a reorientation of the latter. Finally, residue Tyr337 is located at the PH–DH interface, and thus probably contributes to stabilizing the DH domain orientation. The p.Phe78Cys change (and possibly p.Met422Val) can also be included in this group. Substitution of the highly conserved Phe78, which is an unexposed HF residue contributing to a hydrophobic core (involving Leu55, Leu59, Val74, Val133, and Ile137), might structurally perturb the region of the HF domain at its interface with the helical linker. The p.Met422Val change, on the other hand, might perturb the structure of the PH domain, thus perturbing the PH–HF interaction.

The third group of class 1 mutations is formed by residues of the REM domain, interacting with the helical hairpin of the CDC25 domain, which contributes to the allosteric structural switch [Freedman et al., 2006], and whose conformation is essential for both RAS binding to the active site and nucleotide exchange [Hall et al., 2001; Margarit et al., 2003] (class 1C: p.Phe623Ile and p.Tyr702His) (Fig. 2C). A single mutation has been identified to affect Phe623, a highly conserved residue of the REM domain located at the interface with the CDC25 domain. This residue is part of an extended hydrophobic groove of the REM domain that accommodates the side chains of two hydrophobic residues (Ile956 and Phe958) of the helical hairpin of the CDC25 domain. Such a hydrophobic interaction has been demonstrated to be important for the correct orientation of the helical hairpin, and for SOS1's catalytic activity [Hall et al., 2001]. Based on these observations, it can be hypothesized that the p.Phe623Ile substitution might perturb the catalytic activity of the CDC25 domain by acting either on RAS binding at the catalytic site, on the domain's catalytic efficiency or on allosteric control. A similar effect might be associated with substitution of the invariant Tyr702, which is located within the REM domain at the interface with the CDC25 domain, in close proximity to the region of the latter that undergoes the conformational switch promoted by RAS binding at the distal site.

Although the primary anchorage of SOS1 to the plasma membrane is mediated by docking of its C-terminal region to SH3 domain-containing adaptor proteins (e.g., GRB2) that bind to the activated receptors, additional anchorage sites at the membrane are provided by the phosphatidylinositol-4,5-phosphate (PIP_2_)- and phosphatidic acid (PA)-binding sites within the PH domain [Chen et al., 1997; Zhao et al., 2007; Zheng et al., 1997], and by an extended positively charged surface of the HF domain that interacts with anionic membranes, and possibly also binds PA and PIP_2_ [Guerasko et al., 2010; Yadav and Bar-Sagi, 2010]. These sites appear to have complementary roles: whereas those within the PH domain mediate SOS1's targeting to the membrane, the latter are thought to activate the GEF at the membrane. In the autoinhibited structure of SOS1, the positively charged site of the HF domain is not oriented in a way that would allow membrane binding [Guerasko et al., 2010]. This finding suggests the possibility that the reorientation of the PA-binding site of the HF domain might be coupled to the destabilization of the autoinhibited conformation of the protein, permitting SOS1's activation through RAS binding at the allosteric site [Guerasko et al., 2010; Yadav and Bar-Sagi, 2010]. Class 2 mutations include changes that are predicted to enhance SOS1's catalytic function by membrane-dependent mechanisms. Within this class, a first group is predicted to perturb the self-inhibiting orientation of the HF domain by affecting solvent exposed residues located within the positively charged surface of the HF domain, thus favoring its membrane binding (class 2A: p.Pro102Arg and p.Glu108Lys, and probably pathogenetic variant p.Pro112Arg) (Fig. 2D). Significantly, these mutations introduce a positively charged amino acid (arginine or lysine) enhancing the positive electrostatic potential of the HF surface. In particular, Glu108 is adjacent to the PA-binding motif and its substitution by lysine generates a contiguous patch of positively charged residues that was recently demonstrated to potentiate membrane binding [Yadav and Bar-Sagi, 2010]. A similar perturbing effect would be predicted for the c.305C>G and c.335C>G missense changes affecting the closely located Pro102 and Pro112 residues.

A second group includes mutations affecting the membrane-binding surface of the PH domain (class 2B: p.Lys427_Asp430delinsAsn, p.Trp432Arg, p.Trp432_Glu433del, p.Glu433Lys, p.Gly434Arg/Lys, p.Gln477Arg/His, p.Pro478Arg, p.Pro481_Gly482insArgLeuPro, and probably pathogenetic variants p.Glu424Lys, p.Gly482Arg, and p.Leu490Arg) (Fig. 2E). As anticipated, two sites within the PH domain have been identified [Chen et al. 1997; Zhao et al., 2007; Zheng et al., 1997]. Both sites contribute to membrane recruitment of SOS1 and are required for efficient GEF activity. We noticed that the vast majority of mutations affecting the PH domain involved solvent-exposed residues within two adjacent regions (i.e., residues 424–436 and 473–493) that do not overlap spatially with the PIP_2_-binding site, which is constituted by residues Lys456, Arg459, Lys472, and Arg489 [Zheng et al., 1997]. A first cluster of mutations affected residues Gln477, Pro478, Pro481, and Gly482, which are placed within the PA-binding site encompassing residues 472–483 [Zhao et al., 2007], whereas the second cluster affect residues Glu424, Lys427–Asp430, Trp432–Gly434, and Leu490. Of note, the nature of the substitution seemed to be critical because all missense mutations affecting these regions resulted in the introduction of a positively charged residue. In two cases (Gly434, Gln477), multiple lesions were predicted to introduce either an arginine or a lysine, further indicating a specific role for the introduced charged residue. In additional two cases, including the recurrent c.1297G>A (p.Glu433Lys), the positively charged residue replaced a negatively charged amino acid. Similarly, the three identified in-frame indels were predicted to increase the electrostatic potential of the postulated membrane interaction surface. Based on established evidence supporting an absolute requirement of SOS1's recruitment to the plasma membrane for RAS activation in response to an extracellular stimulus [Zhao et al., 2007], this nonrandom distribution and type of lesions strongly suggest that their pathogenetic effect is related to a strengthened binding of SOS1 to PA, PIP_2_ and/or other membrane phospholipids, and consequently, to a more stable recruitment of the protein at the membrane, which in turn would enhance the duration and/or amplitude of GEF function.

Only two disease-causing mutations have been found to affect the CDC25 domain so far. The affected residues (i.e., Glu846 and Pro894) are placed in close proximity to each other, and distally from the two RAS binding sites and regions implicated in the conformational rearrangement of the domain promoting RAS's binding to the active site (Fig. 2F). The high recurrence of the p.Glu846Lys change indirectly documents a relevant role of this region in SOS1's function. This amino acid substitution has recently been documented to profoundly perturb intracellular signaling [Chen et al., 2010]. Available crystal data, however, do not allow us to infer any functional effect for these mutations, as well as for unclassified variants affecting residues in this domain (Ile784, Gln977, and Ser1000) or at the C-terminus (Arg1131, Leu1140, and Thr1257). We noted that Arg1131 is adjacent to Ser1132, which is one of the identified growth factor-induced, MAPK-mediated phosphorylation sites of SOS1 [Corbalan-Garcia et al., 1996]. These sites cluster within a small region that contains the proline-rich SH3-binding sites implicated in GRB2 binding. Of note, phosphorylation of these sites has been demonstrated to reduce SOS1's binding affinity for GRB2, and it could be speculated that mutations altering the recognition motif at these sites might affect phosphorylation and promote enhanced GRB2 binding. Similarly, Leu1140 and Thr1257 cluster within this region. Based on evidence suggesting that this region possibly exerts an autoinhibitory effect on SOS1's activity [Aronheim et al., 1994; Wang et al., 1995], it can be speculated that lesions affecting this region might perturb such an inhibitory mechanism.

Finally, available structural data did not allow us to identify any functional clue for the p.Asp309Tyr NS-causing substitution as well as for the unclassified sequence variants affecting Thr37 and Thr378 residues.

### Phenotypic Spectrum and Genotype–Phenotype Correlations of *SOS1* Mutations

Extensive clinical information was obtained for 39 subjects with NS and bona fide *SOS1* mutations, including individuals recruited in this study (group 1, cases NS01 to NS25) and reexamined subjects of our original study (NS26 to NS39) [Tartaglia et al., 2007] (Supp. Table S3). Overall, analysis of the clinical data confirmed previous observations from our group and others indicating that mutations in *SOS1* are associated with a distinctive phenotype that unambiguously falls within the NS clinical spectrum, but is characterized by a high prevalence of ectodermal features (keratosis pilaris/hyperkeratotic skin, sparse eyebrows, and sparse, generally thin and curly scalp hair) (84% of cases), and a relatively low occurrence of cognitive deficits (11% of cases) compared to what is observed in the NS general population. Of note, in two cases mental retardation was potentially attributable to critical illness during infancy. In two additional subjects, the intelligence quotient was borderline to the lower limit of the normal range with impairment of linguistic skills and oculomanual coordination in one subject, and delay of speech in the other (Supp. Table S3). We also confirmed our previous observation indicating a relatively low prevalence of subjects exhibiting reduced growth (length/stature below the third centile) (29% of cases). In these subjects, reduced growth was invariably associated with delayed bone age. Remarkably, more than one-third of subjects with mutated *SOS1* allele exhibited fetal macrosomia, which however, did not appear to correlate with the extent of their postnatal growth, being length/stature in these subjects below the third centile in a comparable proportion of cases, compared with subjects without this feature (3/14 vs. 7/24, Fisher's exact probability = 0.45). Of note, *SOS1* mutation-positive subjects displayed a more pronounced growth failure (45% of cases) as newborns, which was not generally secondary to poor sucking and/or swallowing.

**Figure 3:**
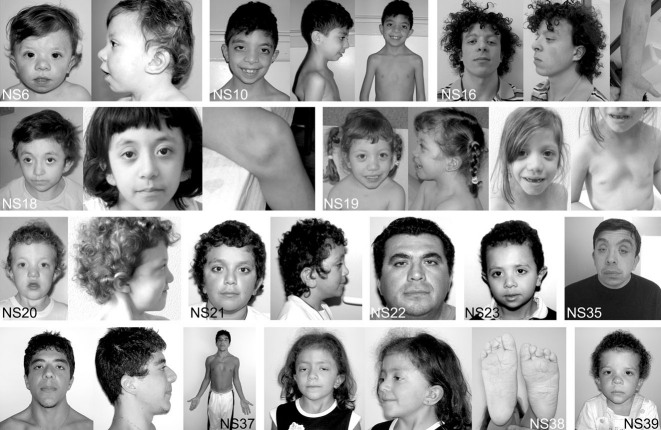
Facial dysmorphism and other features of subjects with Noonan syndrome heterozygous for mutations in the *SOS1* gene. *SOS1* mutation-positive subjects generally exhibit typical facial features, including macrocephaly, hypertelorism, ptosis, downslanting palpebral fissures, sparse eyebrows with keratosis pylaris, a short and broad nose with upturned tip, low-set and posteriorly angulated ears, and high forehead commonly associated with bitemporal narrowing and prominent supraorbital ridges. Curly hair is present in most of the patients. Other common features include pectus anomalies (NS10, NS19, NS37), short and/or webbed neck (NS6, NS10, NS19, NS22, NS38), and cubitus valgus (NS37). Keloid scars (NS16), recurrent hemorrhages (NS18), and deep plantar creases (NS38) also occur in these subjects. In some infants, the face is suggestive of cardiofaciocutaneous syndrome due to the coarseness of features (NS39).

*SOS1* mutation-positive subjects displayed typical facial features (Fig. 3). Macrocephaly was, however, overrepresented compared to the general NS population (61 vs. 12%) [Sharland et al., 1992]. Among these subjects, cardiac defects were frequently observed (89% of cases), with PS, ASD. and VSD being the most recurrent anomalies, while prevalence of HCM was comparable to that observed in *PTPN11* mutation-associated NS, occurring in less than 10% of cases [Sarkozy et al., 2009b]. Of note, PS was found to be frequently associated with ASD (35% of cases). Finally, two subjects were documented to present with mandibular multiple giant cell lesions (MGCLs), which were associated with multiple tumors, including abdominal rhabdomyosarcoma, cerebral glioma, and skin granular cell tumors in one subject.

The analysis of distribution of the major clinical features among *SOS1* mutation-positive subjects documented a significantly higher prevalence of fetal macrosomia in subjects with class 1B mutations compared to individuals with class 1A mutations (Fisher's exact probability = 0.024). We failed in identifying any other significant genotype–phenotype correlation. Based on the relatively small size of the cohort analyzed, however, the occurrence of other associations between specific features and individual *SOS1* lesions or mutation clusters cannot be ruled out.

## Discussion

In this report, we have expanded the available information about the molecular diversity of *SOS1* mutations underlying NS, and have provided a more comprehensive assessment of the clinical features associated with those molecular lesions. We also explored systematically the predicted structural consequences of NS-causing *SOS1* defects, developing a classification of these gene lesions based on the predicted role of affected residues and functional consequences derived from the nature of the amino acid change.

Combined with data from previous surveys, our findings indicate that *SOS1* mutations are almost always missense changes, although we documented that small in-frame indels occur in a small proportion of cases. Available mutation records (data updated to July 2010) revealed a complete absence of nonsense, frameshift, and splicing defects, which is consistent with functional characterization of a panel of NS-causing *SOS1* mutations [Chen et al., 2010; Guerasko et al., 2010; Roberts et al., 2007; Tartaglia et al., 2007], and further support their activating role on SOS1 functional dysregulation in disease pathogenesis. Moreover, the accumulated data strongly indicated that specificity in the amino acid substitution is relevant to the functional dysregulation of the protein. Specifically, occurrence of invariant amino acid changes was observed for several residues and accounted for approximately 50% of total events, strongly suggesting a specific role for the introduced residue. Exemplifying this is the observation of positively charged amino acids that were observed invariantly to replace solvent exposed residues located within the HF and PH domains (i.e., Glu108, Trp432, Glu433, and Gly434), which provides strong evidence for the electrostatic nature of the perturbing effect of those mutations on SOS1 functional dysregulation. On the other hand, the identity of substitution did not seem to be critical in other cases (e.g., most mutations affecting the helical linker connecting the DH and PH domain), indicating a crucial role for the amino acid residue being replaced. This is the case of Arg552, for which all but one amino acid substitutions resulting from a single base change affecting this codon have been documented to cause NS.

Based on SOS1's crystal structure and mechanism of activation [Guerasko et al., 2008, 2010], the nonrandom distribution pattern of altered residues and nature of substitutions indicate that NS-causing mutations dysregulate SOS1's GEF function by at least two major mechanisms. As originally reported by Roberts et al. [2007] and Tartaglia et al. [2007], a large fraction of mutations was found to cluster in regions of the protein that participate in the interdomain binding network that maintain SOS1 in its catalytically inactive conformation (class 1 mutations). Among these, mutations can affect either residues directly implicated in the autoinhibitory interaction between the DH and REM domains that impair GTP-RAS binding at the distal site (or residues closely located to them), or residues located within the DH–PH helical linker or regions of the HF, DH, and PH domains that contribute to stabilizing the HF, DH, and REM interdomain binding network that maintains SOS1 in its catalytically inactive conformation. Of note, this study recognized a third group of class 1 mutations that were found to specifically affect residues of the REM domain located at the interface with regions of the CDC25 domain identified to undergo the GTP-RAS binding-induced conformational rearrangement required for both RAS binding to the active site and nucleotide exchange reaction. Although the effect of these amino acid substitutions on RAS binding at the catalytic site or on the domain's catalytic efficiency cannot be ruled out, we hypothesize that these mutations might upregulate SOS1's catalytic activity by weakening the inhibitory allosteric control on the active site.

Strikingly, our analysis also allowed us to discern a previously unrecognized class of mutations affecting solvent exposed residues located within the membrane oriented surface of the PH domain and a positively charged surface of the HF domain, and predicted to enhance SOS1's catalytic function by distinct membrane-dependent mechanisms. Although the complex process of SOS1's catalytic activation has not been characterized in detail, available structural and functional data indicate that the two major events controlling SOS1's function, that is, protein recruitment to the plasma membrane and release of autoinhibition, are linked. Membrane translocation of SOS1 is promoted by binding of the protein to activated cell surface receptors via SH3 domain recognition sites at the C-terminus of SOS1 that mediate its interaction with adaptor proteins. Additional anchorage sites at the membrane, however, are also provided by the PH and HF domains. Experimental and structural data indicate that two distinct sites within the PH domains bind to PIP_2_ and PA, and contribute significantly to SOS1's targeting to the membrane [Chen et al., 1997; Zhao et al., 2007]. On the other hand, in the SOS1's autoinhibited structure, the positively charged surface of the HF domain is not oriented in a way that would allow membrane binding, suggesting the possibility that reorientation of the HF domain in the membrane-bound conformation might destabilize HF's interaction with the REM domain, unmasking the distal RAS binding site [Guerasko et al., 2010; Yadav and Bar-Sagi, 2010]. Based on this model, the molecular spectrum of disease-causing mutations affecting the PH and HF domains are predicted to favor the membrane-dependent electrostatic switch leading to a productive reorientation of the protein in the plane of the membrane and consequent release of autoinhibition at the regulatory RAS binding site. In this scenario, class 2 mutations would enhance the simultaneous engagement of the membrane by the PIP2- and PA-binding pockets of the PH domain and the positively charged surface of the HF domain, synergizing to increase the stability of SOS1 translocation at the membrane and catalytic activation. Specifically, the pathogenetic effect of this class of mutations affecting the PH domain would be related to a more stable recruitment of the protein at the membrane, while the introduction of an arginine or lysine residue within the positively charged surface of the HF domain would favor a spatial reorientation of the domain weakening its inhibitory function. Consistent with this model, Guerasko and coworkers [2010] demonstrated that the Glu108Lys substitution promotes enhanced SOS1's GEF activity in a PIP_2_-dependent manner, and elegantly provided evidence for a role of nonspecific electrostatic interactions of the HF domain with the membrane in release of autoinhibition and activation of SOS1.

The present mutation scanning of a clinically well-characterized cohort of subjects with features fitting or highly suggestive of NS, and negative for mutations in *PTPN11*, *RAF1*, and *KRAS*, confirmed our previous estimate of *SOS1* mutation prevalence, indicating that defects in this gene account for approximately 10% of NS. Moreover, detailed clinical examination of 39 mutation-positive individuals, including data from clinical reexamination of 14 subjects constituting our original cohort [Tartaglia et al., 2007], provided a more complete assessment of the phenotypic variation associated with these molecular lesions, and strengthened the specific association with ectodermal involvement (84%), and decreased prevalence of cognitive deficits (11%) documented in previous reports [Denayer et al., 2010; Roberts et al., 2007; Tartaglia et al., 2007; Zenker et al., 2007a]. A major finding also regarded the relatively lower prevalence of short stature/length below the third centile in these subjects (29%) compared to the overall NS population, for which an estimate of 70% is generally reported. This percentage is lower than in other studies, in which the prevalence ranged from 31 to 64% [Denayer et al., 2010; Roberts et al., 2007; Zenker et al., 2007a]. Based on the different recruitment strategies utilized, this discrepancy might be due, in part, to differences in patient selection (e.g., recruitment performed by pediatric endocrinology units vs. cardiology units). It should be noted, however, that this disagreement might be also related to a sampling bias depending on the age distribution among cohorts. In NS, although birth length is typically normal, growth parameters usually drop below the third centile during the first years of life. Because there is often some attenuation and/or delay of the pubertal growth spurt, the prevalence of short stature in NS is expected to be highest during the age of normal puberty. Because of the delay in bone age, however, many patients have some catchup growth in their late teens. As we documented a delay in bone age in all subjects with length/stature below the third centile, a significant variation in the estimate of the growth status between cohorts differing considerably in age distribution would be expected, because the estimate of reduced growth prevalence in any NS cohort would be sensitive to the composition of the cohort in terms of proportion of pediatric versus adult subjects.

We observed the occurrence of multiple tumors (i.e., MGCLs, abdominal rhabdomyosarcoma, cerebral glioma, and granular cell tumors of the skin) in one of the *SOS1* mutation-positive subjects included in the study. It has been established that NS patients are at increased risk of developing childhood myeloproliferative disease (i.e., juvenile myelomonocytic leukemia) and acute leukemia [Tartaglia and Gelb, 2005], and that this specific association is strictly linked to a specific class of activating germline mutations in the *PTPN11* gene [Tartaglia et al., 2006]. Occurrence of neuroblastoma, glial tumors, and rhabdomyosarcoma in subjects with NS, however, has also been reported [Kratz, 2009]. Remarkably, although mutations in *SOS1* have previously been considered to be more benign in terms of risk of malignancy than those in other genes in the RAS/MAPK pathway (e.g., *PTPN11*, *HRAS*, *KRAS,* and *NF1*) [Swanson et al., 2008], a significant high occurrence of solid tumors, particularly embryonal rhabdomyosarcoma, has recently been reported in subjects with NS due to a mutated *SOS1* allele [Denayer et al., 2010; Hastings et al., 2010; Jongmans et al., 2010]. The present finding is in line with these recent reports, and provides further evidence that subjects heterozygous for *SOS1* mutations may have a significant risk for certain solid tumors. Of note, MGCLs were observed in one additional *SOS1* mutation-positive subject of the present cohort. MGCLs are benign tumor-like lesions most frequently affecting the jaws but also occurring in other bones or soft tissues. They consist of an osteoblast-like cell population representing the proliferating tumor cells producing cytokines inducing the maturation of a subset of phagocytes into osteoclast-like giant cells [de Lange et al., 2007]. Although the incidence of MGCLs in patients with NS is not known, they were originally linked to a narrow spectrum of germline *PTPN11* mutations [Jafarov et al., 2005; Lee et al., 2005; Tartaglia et al., 2002]. In recent years, however, their co-occurrence in NS (and other RAS-opathies) has been extended to other genes encoding transducers with roles in the RAS–MAPK pathway, with *SOS1* being the most frequently mutated gene [Beneteau et al., 2009; Hanna et al., 2009; Neumann et al., 2009], indicating that SOS1 is a major predisposing gene for MGCLs in NS.

One patient in the present cohort, who was heterozygous for the de novo c.1297G>A missense change (p.Gly434Lys), presented with gingival hypertrophy. Interestingly, a *SOS1* frameshift mutation (c.3248–3249insC) predicting a truncated protein lacking the C-terminal region encompassing the SH3 domain-binding and MAPK phosphorylation sites, was previously documented to be responsible for a form of hereditary gingival fibromatosis (HGF1; MIM♯ 135300), a genetically heterogeneous benign gingival overgrowth condition, in a large family [Hart et al., 2002]. Consistent with the negative modulatory role of the C-terminal region on SOS1 function [Corbalan-Garcia et al., 1998; Wang et al., 1995], the SOS1 mutant was demonstrated to localize at the plasma membrane constitutively and sustain the activation of RAS–MAPK signaling, linking the gingival overgrowth to enhanced SOS1-mediated signal flow through RAS [Jang et al., 2007]. Although further studies are required to appreciate more precisely the peculiar effect of the HGF1-associated mutation on SOS1 functional dysregulation and its specific effect on gingival growth, the present finding provides further evidence in support of a relation between RAS/MAPK signaling dysregulation and gingival hypertrophy.

Excluding Down syndrome, NS is the most frequent developmental disorder associated with congenital cardiac disease. Among *SOS1* mutation-positive individuals, cardiac defects occurred in the majority of cases (>85%), which is consistent with previous studies [Denayer et al., 2010; Roberts et al., 2007; Tartaglia et al., 2007; Zenker et al., 2007a]. Similar to what observed in NS subjects with *PTPN11* mutations [Tartaglia et al., 2002; Zenker et al., 2004], *SOS1* mutation-positive patients exhibit a high prevalence of PS (68% of cases), which was typically associated with ASD (35% of cases), a relatively high occurrence of atrial and ventricular septal defects (39% of cases), and a low prevalence of HCM (<10% of cases). Based on these findings and preliminary observations suggesting that a distinct class of mutations in NS disease might be implicated in isolated CHDs [Greenway et al., 2009; Pandit et al., 2007], and given the relatively “mild” developmental and growth related phenotype documented in a significant proportion of *SOS1* mutation-positive subjects, we evaluated the possible contribution of germline *SOS1* gene lesions in apparently nonsyndromic PS, ASD, and VSD. Mutation analysis revealed no bona fide mutation within these cohorts, which does not support the idea of a major contribution of *SOS1* gene defects in the pathogenesis of these CHDs, in line with other studies documenting only a minor contribution of germline *PTPN11* mutations in isolated CHDs and HCM [Limongelli et al., 2006; Roberts et al., 2005; Sarkozy et al., 2003, 2005; Weismann et al., 2005].

Subjects heterozygous for a mutated *SOS1* allele present with ectodermal manifestations and distinctive facial dysmorphism that might be suggestive of CFCS in some individuals (Fig. 3) [Narumi et al., 2008; Nystrom et al., 2008]. In these subjects, however, cognitive deficits are generally absent or minor [Denayer et al., 2010; Narumi et al., 2008; Tartaglia et al., 2007; Zenker et al., 2007a; present study]. Consistent with these observations, our mutational screening on a clinically well-characterized CFCS cohort failed in identifying any *SOS1* mutation, confirming a previous survey indicating that *SOS1* does not represent a major gene for this disorder [Zenker et al. 2007a]. Atlhough the clinical features of *SOS1* mutation-positive subjects appear to be less severe compared to what is generally observed in CFCS, the identification of subjects with an “overlapping” phenotype suggests that a clinical continuum might be associated with defects in *SOS1*, as previously documented for other disease genes implicated in RAS-opathies [Sarkozy et al., 2009a; Zenker et al., 2007b]. On the other hand, based on the increasing evidence documenting co-occurrence of mutations in functionally related genes that contribute to the severity of the phenotype [Longoni et al., 2010; Nystrom et al., 2009; Tang et al., 2009; Thiel et al., 2009], the possibility that mutations concomitant to those affecting *SOS1* might modulate the phenotype in these subjects cannot be ruled out. Overall, these findings further emphasize the difficulty in identifying efficient clinical criteria to define these disorders nosologically, and make evident the clinical relevance of a molecular-based definition of these clinically overlapping disorders.

## References

[b1] Allanson JE (1987). Noonan syndrome. J Med Genet.

[b2] Aoki Y, Niihori T, Narumi Y, Kure S, Matsubara Y (2008). The RAS/MAPK syndromes: novel roles of the RAS pathway in human genetic disorders. Hum Mutat.

[b3] Aronheim A, Engelberg D, Li N, al-Alawi N, Schlessinger J, Karin M (1994). Membrane targeting of the nucleotide exchange factor Sos is sufficient for activating the Ras signaling pathway. Cell.

[b4] Baker NA, Sept D, Joseph S, Holst MJ, McCammon JA (2001). Electrostatics of nanosystems: application to microtubules and the ribosome. Proc Natl Acad Sci USA.

[b5] Beneteau C, Cave H, Moncla A, Dorison N, Munnich A, Verloes A, Leheup B (2009). SOS1 and PTPN11 mutations in five cases of Noonan syndrome with multiple giant cell lesions. Eur J Hum Genet.

[b6] Burch M, Sharland M, Shinebourne E, Smith G, Patton M, McKenna W (1993). Cardiologic abnormalities in Noonan syndrome: phenotypic diagnosis and echocardiographic assessment of 118 patients. J Am Coll Cardiol.

[b7] Chen PC, Wakimoto H, Conner D, Araki T, Yuan T, Roberts A, Seidman CE, Bronson R, Neel BG, Seidman JG, Kucherlapati R (2010). Activation of multiple signaling pathways causes developmental defects in mice with a Noonan syndrome-associated Sos1 mutation. J Clin Invest.

[b8] Chen RH, Corbalan-Garcia S, Bar-Sagi D (1997). The role of the PH domain in the signal-dependent membrane targeting of Sos. EMBO J.

[b9] Corbalan-Garcia S, Yang SS, Degenhardt KR, Bar-Sagi D (1996). Identification of the mitogen-activated protein kinase phosphorylation sites on human Sos1 that regulate interaction with Grb2. Mol Cell Biol.

[b10] Corbalan-Garcia S, Margarit SM, Galron D, Yang SS, Bar-Sagi D (1998). Regulation of Sos activity by intramolecular interactions. Mol Cell Biol.

[b11] de Lange J, van den Akker HP, van den Berg H (2007). Central giant cell granuloma of the jaw: a review of the literature with emphasis on therapy options. Oral Surg Oral Med Oral Pathol Oral Radiol Endod.

[b12] Denayer E, Devriendt K, de Ravel T, Van Buggenhout G, Smeets E, Francois I, Sznajer Y, Craen M, Leventopoulos G, Mutesa L, Vandecasseye W, Massa G, Kayserili H, Sciot R, Fryns JP, Legius E (2010). Tumor spectrum in children with Noonan syndrome and SOS1 or RAF1 mutations. Genes Chromosomes Cancer.

[b13] Ferrero GB, Baldassarre G, Delmonaco AG, Biamino E, Carta C, Rossi C, Silengo MC (2008). Clinical and molecular characterization of 40 patients with Noonan syndrome. Eur J Med Genet.

[b14] Freedman TS, Sondermann H, Friedland GD, Kortemme T, Bar-Sagi D, Marqusee S, Kuriyan J (2006). A Ras-induced conformational switch in the Ras activator Son of sevenless. Proc Natl Acad Sci USA.

[b15] Greenway SC, Pereira AC, Lin JC, DePalma SR, Israel SJ, Mesquita SM, Ergul E, Conta JH, Korn JM, McCarroll SA, Gorham JM, Gabriel S, Altshuler DM, Quintanilla-Dieck Mde L, Artunduaga MA, Eavey RD, Plenge RM, Shadick NA, Weinblatt ME, De Jager PL, Hafler DA, Breitbart RE, Seidman JG, Seidman CE (2009). De novo copy number variants identify new genes and loci in isolated sporadic tetralogy of Fallot. Nat Genet.

[b16] Guerasko J, Galush WJ, Boykevisch S, Sondermann H, Bar-Sagi D, Groves JT, Kuriyan J (2008). Membrane-dependent signal integration by the Ras activator Son of sevenless. Nat Struct Mol Biol.

[b17] Guerasko J, Kuchment O, Makino DL, Sondermann H, Bar-Sagi D, Kuriyan J (2010). Role of the histone domain in the autoinhibition and activation of the Ras activator Son of Sevenless. Proc Natl Acad Sci USA.

[b18] Hall BE, Yang SS, Boriack-Sjodin PA, Kuriyan J, Bar-Sagi D (2001). Structure-based mutagenesis reveals distinct functions for Ras switch 1 and switch 2 in Sos-catalyzed guanine nucleotide exchange. J Biol Chem.

[b19] Hanna N, Parfait B, Talaat IM, Vidaud M, Elsedfy HH (2009). SOS1: a new player in the Noonan-like/multiple giant cell lesion syndrome. Clin Genet.

[b20] Hart TC, Zhang Y, Gorry MC, Hart PS, Cooper M, Marazita ML, Marks JM, Cortelli JR, Pallos D (2002). A mutation in the SOS1 gene causes hereditary gingival fibromatosis type 1. Am J Hum Genet.

[b21] Hastings R, Newbury-Ecob R, Ng A, Taylor R (2010). A further patient with Noonan syndrome due to a SOS1 mutation and rhabdomyosarcoma. Genes Chromosomes Cancer.

[b22] Jafarov T, Ferimazova N, Reichenberger E (2005). Noonan-like syndrome mutations in PTPN11 in patients diagnosed with cherubism. Clin Genet.

[b23] Jang SI, Lee EJ, Hart PS, Ramaswami M, Pallos D, Hart TC (2007). Germ line gain of function with SOS1 mutation in hereditary gingival fibromatosis. J Biol Chem.

[b24] Jongmans MC, Hoogerbrugge PM, Hilkens L, Flucke U, van der Burgt I, Noordam K, Ruiterkamp-Versteeg M, Yntema HG, Nillesen WM, Ligtenberg MJ, van Kessel AG, Kuiper RP, Hoogerbrugge N (2010). Noonan syndrome, the SOS1 gene and embryonal rhabdomyosarcoma. Genes Chromosomes Cancer.

[b25] Ko JM, Kim JM, Kim GH, Yoo HW (2008). PTPN11, SOS1, KRAS, and RAF1 gene analysis, and genotype–phenotype correlation in Korean patients with Noonan syndrome. J Hum Genet.

[b26] Kratz C, Zenker M (2009). Myeloproliferative disease and cancer in persons with Noonan syndromeand related disorders. Monographs of Human Genetic Noonan Syndrome and Related Disorders.

[b27] Lee JS, Tartaglia M, Gelb BD, Fridrich K, Sachs S, Stratakis CA, Muenke M, Robey PG, Collins MT, Slavotinek A (2005). Phenotypic and genotypic characterisation of Noonan-like/multiple giant cell lesion syndrome. J Med Genet.

[b28] Limongelli G, Hawkes L, Calabro R, McKenna WJ, Syrris P (2006). Mutation screening of the PTPN11 gene in hypertrophic cardiomyopathy. Eur J Med Genet.

[b29] Longoni M, Moncini S, Cisternino M, Morella IM, Ferraiuolo S, Russo S, Mannarino S, Brazzelli V, Coi P, Zippel R, Venturin M, Riva P (2010). Noonan syndrome associated with both a new Jnk-activating familial SOS1 and a de novo RAF1 mutations. Am J Med Genet A.

[b30] Margarit SM, Sondermann H, Hall BE, Nagar B, Hoelz A, Pirruccello M, Bar-Sagi D, Kuriyan J (2003). Structural evidence for feedback activation by Ras.GTP of the Ras-specific nucleotide exchange factor SOS. Cell.

[b31] Marino B, Digilio MC, Toscano A, Giannotti A, Dallapiccola B (1999). Congenital heart diseases in children with Noonan syndrome: an expanded cardiac spectrum with high prevalence of atrioventricular canal. J Pediatr.

[b32] Narumi Y, Aoki Y, Niihori T, Sakurai M, Cave H, Verloes A, Nishio K, Ohashi H, Kurosawa K, Okamoto N, Kawame H, Mizuno S, Kondoh T, Addor MC, Coeslier-Dieux A, Vincent-Delorme C, Tabayashi K, Aoki M, Kobayashi T, Guliyeva A, Kure S, Matsubara Y (2008). Clinical manifestations in patients with SOS1 mutations range from Noonan syndrome to CFC syndrome. J Hum Genet.

[b33] Neumann TE, Allanson J, Kavamura I, Kerr B, Neri G, Noonan J, Cordeddu V, Gibson K, Tzschach A, Kruger G, Hoeltzenbein M, Goecke TO, Kehl HG, Albrecht B, Luczak K, Sasiadek MM, Musante L, Laurie R, Peters H, Tartaglia M, Zenker M, Kalscheuer V (2009). Multiple giant cell lesions in patients with Noonan syndrome and cardio-facio-cutaneous syndrome. Eur J Hum Genet.

[b34] Newcombe RG (1998). Two-sided confidence intervals for the single proportion: comparison of seven methods. Stat Med.

[b35] Ng PC, Henikoff S (2001). Predicting deleterious amino acid substitutions. Genome Res.

[b36] Nimnual A, Bar-Sagi D (2002). The two hats of SOS. Sci STKE.

[b37] Noonan JA (1994). Noonan syndrome. An update and review for the primary pediatrician. Clin Pediatr (Phila).

[b38] Nystrom AM, Ekvall S, Berglund E, Bjorkqvist M, Braathen G, Duchen K, Enell H, Holmberg E, Holmlund U, Olsson-Engman M, Annerén G, Bondeson ML (2008). Noonan and cardio-facio-cutaneous syndromes: two clinically and genetically overlapping disorders. J Med Genet.

[b39] Nystrom AM, Ekvall S, Strömberg B, Holmström G, Thuresson AC, Annerén G, Bondeson ML (2009). A severe form of Noonan syndrome and autosomal dominant café-au-lait spots—evidence for different genetic origins. Acta Paediatr.

[b40] Pandit B, Sarkozy A, Pennacchio LA, Carta C, Oishi K, Martinelli S, Pogna EA, Schackwitz W, Ustaszewska A, Landstrom A, Bos JM, Ommen SR, Esposito G, Lepri F, Faul C, Mundel P, López Siguero JP, Tenconi R, Selicorni A, Rossi C, Mazzanti L, Torrente I, Marino B, Digilio MC, Zampino G, Ackerman MJ, Dallapiccola B, Tartaglia M, Gelb BD (2007). Gain-of-function RAF1 mutations cause Noonan and LEOPARD syndromes with hypertrophic cardiomyopathy. Nat Genet.

[b41] Pettersen EF, Goddard TD, Huang CC, Couch GS, Greenblatt DM, Meng EC, Ferrin TE (2004). UCSF Chimera—a visualization system for exploratory research and analysis. J Comput Chem.

[b42] Roberts AE, Araki T, Swanson KD, Montgomery KT, Schiripo TA, Joshi VA, Li L, Yassin Y, Tamburino AM, Neel BG, Kucherlapati RS (2007). Germline gain-of-function mutations in SOS1 cause Noonan syndrome. Nat Genet.

[b43] Roberts AE, Hult B, Rehm HL, Rehm HL, McDonough B, Barr S, Seidman CE, Seidman JG, Kucherlapati RS (2005). The PTPN11 gene is not implicated in nonsyndromic hypertrophic cardiomyopathy. Am J Med Genet A.

[b44] Sarkozy A, Carta C, Moretti S, Zampino G, Digilio MC, Pantaleoni F, Scioletti AP, Esposito G, Cordeddu V, Lepri F, Petrangeli V, Dentici ML, Mancini GM, Selicorni A, Rossi C, Mazzanti L, Marino B, Ferrero GB, Silengo MC, Memo L, Stanzial F, Faravelli F, Stuppia L, Puxeddu E, Gelb BD, Dallapiccola B, Tartaglia M (2009a). Germline BRAF mutations in Noonan, LEOPARD, and cardiofaciocutaneous syndromes: molecular diversity and associated phenotypic spectrum. Hum Mutat.

[b45] Sarkozy A, Conti E, Esposito G, Pizzuti A, Dallapiccola B, Mingarelli R, Marino B, Digilio MC, Paoletti V (2003). Nonsyndromic pulmonary valve stenosis and the PTPN11 gene. Am J Med Genet A.

[b46] Sarkozy A, Conti E, Lepri FR, Pizzuti A, Dallapiccola B, Autore C, Tartaglia M (2005). Hyperthrophic cardiomyopathy and the PTPN11 gene. Am J Med Genet A.

[b47] Sarkozy A, Digilio MC, Marino B, Dallapiccola B, Zenker M (2009b). Genotype–phenotype correlations in Noonan syndrome. Monographs Human Genetic Noonan Syndrome and Related Disorders.

[b48] Sharland M, Burch M, McKenna WM, Paton MA (1992). A clinical study of Noonan syndrome. Arch Dis Child.

[b49] Sondermann H, Nagar B, Bar-Sagi D, Kuriyan J (2005). Computational docking and solution x-ray scattering predict a membrane-interacting role for the histone domain of the Ras activator son of sevenless. Proc Natl Acad Sci USA.

[b50] Sondermann H, Soisson SM, Boykevisch S, Yang SS, Bar-Sagi D, Kuriyan J (2004). Structural analysis of autoinhibition in the Ras activator Son of sevenless. Cell.

[b51] Sunyaev S, Ramensky V, Bork P (2000). Towards a structural basis of human non-synonymous single nucleotide polymorphisms. Trends Genet.

[b52] Swanson KD, Winter JM, Reis M, Bentires-Alj M, Greulich H, Grewal R, Hruban RH, Yeo CJ, Yassin Y, Iartchouk O, Montgomery K, Whitman SP, Caligiuri MA, Loh ML, Gilliland DG, Look AT, Kucherlapati R, Kern SE, Meyerson M, Neel BG (2008). SOS1 mutations are rare in human malignancies: implications for Noonan Syndrome patients. Genes Chromosomes Cancer.

[b53] Tang S, Hoshida H, Kamisago M, Yagi H, Momma K, Matsuoka R (2009). Phenotype–genotype correlation in a patient with co-occurrence of Marfan and LEOPARD syndromes. Am J Med Genet A.

[b54] Tartaglia M, Gelb BD (2005). Noonan syndrome and related disorders: genetics and pathogenesis. Annu Rev Genomics Hum Genet.

[b55] Tartaglia M, Kalidas K, Shaw A, Song X, Musat DL, van der Burgt I, Brunner HG, Bertola DR, Crosby A, Ion A, Kucherlapati RS, Jeffery S, Patton MA, Gelb BD (2002). PTPN11 mutations in Noonan syndrome: molecular spectrum, genotype–phenotype correlation, and phenotypic heterogeneity. Am J Hum Genet.

[b56] Tartaglia M, Mehler EL, Goldberg R, Zampino G, Brunner HG, Kremer H, van der Burgt I, Crosby AH, Ion A, Jeffery S, Kalidas K, Patton MA, Kucherlapati RS, Gelb BD (2001). Mutations in PTPN11, encoding the protein tyrosine phosphatase SHP-2, cause Noonan syndrome. Nat Genet.

[b57] Tartaglia M, Martinelli S, Stella L, Bocchinfuso G, Flex E, Cordeddu V, Zampino G, Burgt I, Palleschi A, Petrucci TC, Sorcini M, Schoch C, Foa R, Emanuel PD, Gelb BD (2006). Diversity and functional consequences of germline and somatic PTPN11 mutations in human disease. Am J Hum Genet.

[b58] Tartaglia M, Pennacchio LA, Zhao C, Yadav KK, Fodale V, Sarkozy A, Pandit B, Oishi K, Martinelli S, Schackwitz W, Martin J, Bristow J, Carta C, Lepri F, Neri C, Vasta I, Gibson K, Curry CJ, Siguero JP, Digilio MC, Zampino G, Dallapiccola B, Bar-Sagi D, Gelb BD (2007). Gain-of-function SOS1 mutations cause a distinctive form of Noonan syndrome. Nat Genet.

[b59] Tartaglia M, Zampino G, Gelb BD (2010). Noonan syndrome: clinical aspects and molecular pathogenesis. Mol Syndromol.

[b60] Tartaglia M, Gelb BD, Zenker M (2011). Noonan syndrome and clinically related disorders. Best Pract Res Clin Endocrinol Metab.

[b61] Thiel C, Wilken M, Zenker M, Sticht H, Fahsold R, Gusek-Schneider GC, Rauch A (2009). Independent NF1 and PTPN11 mutations in a family with neurofibromatosis-Noonan syndrome. Am J Med Genet A.

[b62] Tidyman WE, Rauen KA (2009). The RASopathies: developmental syndromes of Ras/MAPK pathway dysregulation. Curr Opin Genet Dev.

[b63] van der Burgt I (2007). Noonan syndrome. Orphanet J Rare Dis.

[b64] van der Burgt I, Berends E, Lommen E, van Beersum S, Hamel B, Mariman E (1994). Clinical and molecular studies in a large Dutch family with Noonan syndrome. Am J Med Genet.

[b65] Wang W, Fisher EM, Jia Q, Dunn JM, Porfiri E, Downward J, Egan SE (1995). The Grb2 binding domain of mSos1 is not required for downstream signal transduction. Nat Genet.

[b66] Weismann CG, Hager A, Kaemmerer H, Maslen CL, Morris CD, Schranz D, Kreuder J, Gelb BD (2005). PTPN11 mutations play a minor role in isolated congenital heart disease. Am J Med Genet A.

[b67] Wilson EB (1927). Probable inference, the law of succession, and statistical inference. J Am Stat Assoc.

[b68] Yadav KK, Bar-Sagi D (2010). Allosteric gating of Son of sevenless activity by the histone domain. Proc Natl Acad Sci USA.

[b69] Zenker M, Buheitel G, Rauch R, Koenig R, Bosse K, Kress W, Tietze HU, Doerr HG, Hofbeck M, Singer H, Reis A, Rauch A (2004). Genotype–phenotype correlations in Noonan syndrome. J Pediatr.

[b70] Zenker M, Horn D, Wieczorek D, Allanson J, Pauli S, van der Burgt I, Doerr HG, Gaspar H, Hofbeck M, Gillessen-Kaesbach G, Koch A, Meinecke P, Mundlos S, Nowka A, Rauch A, Reif S, von Schnakenburg C, Seidel H, Wehner LE, Zweier C, Bauhuber S, Matejas V, Kratz CP, Thomas C, Kutsche K (2007a). SOS1 is the second most common Noonan gene but plays no major role in cardio-facio-cutaneous syndrome. J Med Genet.

[b71] Zenker M, Lehmann K, Schulz AL, Barth H, Hansmann D, Koenig R, Korinthenberg R, Kreiss-Nachtsheim M, Meinecke P, Morlot S, Mundlos S, Quante AS, Raskin S, Schnabel D, Wehner LE, Kratz CP, Horn D, Kutsche K (2007b). Expansion of the genotypic and phenotypic spectrum in patients with KRAS germline mutations. J Med Genet.

[b72] Zhao C, Du G, Skowronek K, Frohman MA, Bar-Sagi D (2007). Phospholipase D2-generated phosphatidic acid couples EGFR stimulation to Ras activation by Sos. Nat Cell Biol.

[b73] Zheng J, Chen RH, Corblan-Garcia S, Cahill SM, Bar-Sagi D, Cowburn D (1997). The solution structure of the pleckstrin homology domain of human SOS1. A possible structural role for the sequential association of diffuse B cell lymphoma and pleckstrin homology domains. J Biol Chem.

