# Supplementary material for: SOS1 Mutations in Noonan Syndrome: Molecular Spectrum, Structural Insights on Pathogenic Effects, and Genotype–Phenotype Correlations
Source: Hum Mutat. 2011 Mar 8;32(7):760–72. doi: 10.1002/humu.21492 (PMC3118925; doi:10.1002/humu.21492)
Supplement: Supplementary file 1 [file humu0032-0760-SD1.pdf]

Supp. Table S1. List of disease-unrelated *SOS1* sequence variants identified in the study

Location	Nucleotide change	Amino acid change
Intron 2	c.87+86delG	-
Intron 4	c.345+52C>G	-
Intron 5	c.510+24T>C	-
	c.510+59C>G	-
Intron 6	c.720+25C>G	-
	c.721-69T>C	-
	c.721-18G>C	-
Intron 7	c.864+23C>T	-
	c.864+24G>A	-
Intron 9	c.1074+5G>C	-
	c.1075-25C>G	-
Intron 10	c.1202+25A>T	-
	c.1203-20T>C	-
Exon 11	c.1230G>A	Silent change (Gln410)
	c.1705C>G	p.Leu569Val
	c.1800T>A	silent change (Ile600)
	c.1854C>G	silent change (Tyr618)
Intron 11	c.1859-40T>C	-
	c.1859-37T>C	-
Exon 13	c.1964C>T	p.Pro655Leu
Exon 14	c.2122G>A	p.Ala708Thr
Intron 16	c.2511-98C>G	-
Intron 17	c.2673+14T>C	-
	c.2674-22C>G	-
Exon 18	c.2760G>A	silent change (Arg920)
Intron 18	c.2791+53C>T	-
Intron 19	c.2964+43C>A	-
Exon 20	c.3032A>G	p.Asn1011Ser
Exon 21	c.3093T>C	silent change (Tyr1031)
Intron 21	c.3346+72+73delCT	-
	c.3347-143T>C	-
	c.3347-127T>C	-
Exon 22	c.3357C>T	silent change (Thr1119)
Intron 22	c.3391+7A>G	-
	c.3392-89T>G	-
Exon 24	c.3959A>G	p.His1320Arg

Nucleotide numbering of the exonic variants reflects cDNA numbering with 1 corresponding to the A of the ATG translation initiation codon in the reference sequence (NM_005633.3). Exon 2 corresponds to the first protein coding exon. Position of intronic variants is numbered according to the reference genomic sequence (NG_007530.1).

Supp. Table S2. List of *SOS1* exonic indels and missense changes (updated to July 2010)

Nucleotide change	Amino acid change	Status and functional class	N	References
c.109A>G	p.Thr37Ala	Unclassified variant	1	*p.s.*
c.233T>G	p.Phe78Cys	Probably pathogenic, 1B	1	3
c.305C>G	p.Pro102Arg	Mutation, 2A	1	11
c.322G>A	p.Glu108Lys	Mutation, 2A	4	2, *p.s.*
c.335C>G	p.Pro112Arg	Probably pathogenic, 2A	1	*p.s.*
c.508A>G	p.Lys170Glu	Mutation, 1B	5	6, 11, *p.s.*
c.755T>C	p.Ile252Thr	Probably pathogenic, 1A	1	*p.s.*
c.797C>A	p.Thr266Lys	Mutation, 1A	8	1, 6, 7, 11, *p.s.*
c.806T>C	p.Met269Thr	Mutation, 1A	9	3, 6, 11, *p.s.*
c.806T>G	p.Met269Arg	Mutation, 1A	8	1, 2, 3, 6, 8, *p.s.*
c.925G>T	p.Asp309Tyr	Mutation, uncharacterized	3	1, 3, 5
c.1010A>G	p.Tyr337Cys	Mutation, 1B	1	1
c.1132A>G	p.Thr378Ala	Unclassified variant	1	11
c.1264A>G	p.Met422Val	Probably pathogenic, 1B	1	*p.s.*
c.1270G>A	p.Glu424Lys	Probably pathogenic, 2B	1	*p.s.*
c.1281_1289delGAATAT TGA	p.Lys427_Asp430de linsAsn	Mutation, 2B	1	*p.s.*
c.1294T>C	p.Trp432Arg	Mutation, 2B	5	2, 3, 10, *p.s.*
c.1294_1299delTGGGAG	p.Trp432_Glu433del	Mutation, 2B	1	*p.s.*
c.1297G>A	p.Glu433Lys	Mutation, 2B	14	2, 3, 4, 6, 8, 11, *p.s.*
c.1300G>C	p.Gly434Arg	Mutation, 2B	1	1
c.1300G>A	p.Gly434Arg	Mutation, 2B	3	3, *p.s.*
c.1300_1301delGGinsAA	p.Gly434Lys	Mutation, 2B	1	*p.s.*
c.1310T>C	p.Ile437Thr	Mutation, 1B	2	*p.s.*
c.1322G>A	p.Cys441Tyr	Mutation, 1B	4	6, *p.s.*
c.1430A>G	p.Gln477Arg	Mutation, 2B	2	*p.s.*
c.1431G>T; 1433C>T	p.Gln477His; Pro478Leu	Mutation, 2B	1	3
c.1433C>G	p.Pro478Arg	Mutation, 2B	2	3, *p.s.*
c.1433C>T	p.Pro478Leu	Unclassified variant (2B)	1	*p.s.*
c.1442_1443insAAGACT TCC	p.Pro481_Gly482 insArgLeuPro	Mutation, 2B	1	5

Nucleotide change	Amino acid change	Status and functional class	N	References
c.1444G>C	p.Gly482Arg	Probably pathogenic, 2B	1	*p.s.*
c.1469T>G	p.Leu490Arg	Probably pathogenic, 2B	1	*p.s.*
c.1490G>A	p.Arg497Gln	Probably pathogenic, 1B	1	*p.s.*
c.1642A>C	p.Ser548Arg	Mutation, 1B	8	1, 2, 6, *p.s.*
c.1646C>A	p.Thr549Lys	Probably pathogenic, 1B	1	*p.s.*
c.1649T>C	p.Leu550Pro	Mutation, 1B	2	2, 8
c.1654A>G	p.Arg552Gly	Mutation, 1B	29	1, 2, 3, 8, 11, *p.s.*
c.1655G>C	p.Arg 552Thr	Mutation, 1B	2	9, *p.s.*
c.1655G>T	p.Arg 552Met	Mutation, 1B	2	*p.s.*
c.1655G>A	p.Arg 552Lys	Mutation, 1B	7	2, 3, *p.s.*
c.1656G>C	p.Arg 552Ser	Mutation, 1B	15	2, 6, 9, *p.s.*
c.1656G>T	p.Arg 552Ser	Mutation, 1B	4	3, 5, 8
c.1660_1673delCTTGAT GTAACAATinsAA	p.Leu554_Met558 delinsLys	Mutation, 1B	1	*p.s.*
c.1705C>G	p.Leu569Val	Polymorphism		11, *p.s.*
c.1867 T>A	p.Phe623Ile	Mutation, 1C	1	3
c.1964C>T	p.Pro655Leu	Polymorphism		1, 2, 3, 11, *p.s.*
c.2104T>C	p.Tyr702His	Mutation, 1C	2	2, 3
c.2122G>A	p.Ala708Thr	Polymorphism		*p.s.*
c.2183A>T	p.Lys728Ile	Mutation, 1A	1	12
c.2186G>T	p.Trp729Leu	Mutation, 1A	1	2
c.2197A>T	p.Ile733Phe	Mutation, 1A	2	2, *p.s.*
c.2351T>C	p.Ile784Thr	Unclassified variant	1	*p.s.*
c.2536G>A	p.Glu846Lys	Mutation, 3	19	1, 2, 3, 5, 8, *p.s.*
c.2681C>G	p.Pro894Arg	Mutation, 3	1	*p.s.*
c.2930A>G	p.Gln977Arg	Unclassified variant	1	2
c.2999G>A	p.Ser1000Asn	Unclassified variant	1	3
c.3032A>G	p.Asn1011Ser	Polymorphism		*p.s.*
c.3392G>A	p.Arg1131Lys	Unclassified variant	1	*p.s.*
c.3418T>A	p.Leu1140Ile	Unclassified variant	1	*p.s.*
c.3769A>G	p.Thr1257Ala	Unclassified variant	1	*p.s.*
c.3959A>G	p.His1320Arg	Polymorphism	1	2, *p.s.*

Nucleotide numbering of the exonic variants reflects cDNA numbering with 1 corresponding to the A of the ATG translation initiation codon in the reference sequence (NM_005633.3). *p.s.*, present study.

References

Beneteau C, Cave H, Moncla A, Munnich A, Verloes A, Leheup B. 2009. SOS1 and PTPN11 mutations in five cases of Noonan syndrome with multiple giant cell lesions. Eur J Hum Genet 17:1216-21.

Denayer E, Devriendt K, de Ravel T, Van Buggenhout G, Smeets E, Francois I, Sznajer Y, Craen M, Leventopoulos G, Mutesa L et al. 2010. Tumor spectrum in children with Noonan syndrome and SOS1 or RAF1 mutations. Genes Chromosomes Cancer 49:242-52.

Ferrero GB, Baldassarre G, Delmonaco AG, Biamino E, Carta C, Rossi C, Silengo MC. 2008. Clinical and molecular characterization of 40 patients with Noonan syndrome. Eur J Med Genet 51:566-72.

Hanna N, Parfait B, Talaat IM, Vidaud M, Elsedfy HH. 2009. SOS1: a new player in the Noonan-like/multiple giant cell lesion syndrome.Clin Genet 75: 568–71.

Jongmans MC, Hoogerbrugge PM, Hilkens L, Flucke U, van der Burgt I, Noordam K, Ruiterkamp-Versteeg M, Yntema HG, Nillesen WM, Ligtenberg MJ et al. 2010. Noonan syndrome, the SOS1 gene and embryonal rhabdomyosarcoma. Genes Chromosomes Cancer 49:635-41.

Ko JM, Kim JM, Kim GH, Yoo HW. 2008. PTPN11, SOS1, KRAS, and RAF1 gene analysis, and genotype-phenotype correlation in Korean patients with Noonan syndrome. J Hum Genet 53:999-1006.

Narumi Y, Aoki Y, Niihori T, Sakurai M, Cave H, Verloes A, Nishio K, Ohashi H, Kurosawa K, Okamoto N et al. 2008. Clinical manifestations in patients with SOS1 mutations range from Noonan syndrome to CFC syndrome. J Hum Genet 53:834-41.

Neumann TE, Allanson J, Kavamura I, Kerr B, Neri G, Noonan J, Cordeddu V, Gibson K, Tzschach A, Kruger G et al. 2009. Multiple giant cell lesions in patients with Noonan syndrome and cardio-facio-cutaneous syndrome. Eur J Hum Genet 17:420-5.

Nystrom AM, Ekvall S, Berglund E, Bjorkqvist M, Braathen G, Duchen K, Enell H, Holmberg E, Holmlund U, Olsson-Engman M et al. 2008. Noonan and cardio-facio-cutaneous syndromes: two clinically and genetically overlapping disorders. J Med Genet 45:500-6.

Roberts AE, Araki T, Swanson KD, Montgomery KT, Schiripo TA, Joshi VA, Li L, Yassin Y, Tamburino AM, Neel BG et al. 2007. Germline gain-of-function mutations in SOS1 cause Noonan syndrome. Nat Genet 39:70-4.

Tartaglia M, Pennacchio LA, Zhao C, Yadav KK, Fodale V, Sarkozy A, Pandit B, Oishi K, Martinelli S, Schackwitz W et al. 2007. Gain-of-function SOS1 mutations cause a distinctive form of Noonan syndrome. Nat Genet 39:75-9.

Zenker M, Horn D, Wieczorek D, Allanson J, Pauli S, van der Burgt I, Doerr HG, Gaspar H, Hofbeck M, Gillessen-Kaesbach G et al. 2007. SOS1 is the second most common Noonan gene but plays no major role in cardio-facio-cutaneous syndrome. J Med Genet 44:651-6.

Supp.Table S3. Clinical features of *SOS1* mutation-positive subjects

**Case**	**NS1**	**NS2**	**NS3**	**NS4**	**NS5**	**NS6**	**NS7**	**NS8**	**NS9**	**NS10**
Code	S1021	S1036	S1052	BO-2564	BO-0138	BO-4043	BO-1767	BO-2905	BO-0235	BO-1834
Sex	M	M	F	M	M	M	M	M	M	M
Age at evaluation	11 y	12 m	1 y	12 m	8 y, 10 m	5 m, 13 d	2 y, 1 m	14 y, 5 m	4 y, 2 m	8 y, 2 m
Amino acid change	R552K	W432_E433del	R552S	E433K	R552G	E433K	T266K	P478R	M269T	M269T
Polyhydramnios	+	-	+	+	-	-	+	-	-	+
Fetal macrosomia	-	-	+	+	-	+	-	-	-	-
Neonatal/infantile growth failure				-	+	-	-	-	+	+
Poor sucking	-	-	+	-	-	-	-	-	+	-
Poor swallowing	-	-	+	-	-	-	-	-	-	-
Apneas	-	-	-	+	-	-			-	-
Short stature (<3rd centile)	-	-	-	-	-	-	-	+	-	+
Height/length (cm)				71	127	65	98	147	105	113
Age				12 m	8 y, 10 m	5 m, 13 d	2 y, 1 m	14 y, 5 m	4 y, 2 m	8 y, 2 m
Centile	5th	50th	75th	5th – 10th	25th – 50th	25th – 50th	>98th	<2nd	50th	<2nd
Delayed bone age								+		+
GH deficiency					+			+		
Craniofacial anomalies	+	+	+	+	+	+	+	+	+	+
- Macrocephaly	-	-	-	+	+	-	+	-	-	-
- Scaphocephaly	-	-	-	-	-	-	-	-	-	-
- High forehead	+	+	-	-	+	+	+	+	+	+
- Bitemporal narrowing	+	+	-	-	+	+	+	-	+	+
- Prominent supraorbital ridge	-	-	-	-	+	+	+	+	-	+
- Downslanting palpebral fissures	+	-	+	+	+	+	+	+	+	+
- Hypertelorism	-	-	+	+	+	+	+	-	+	+
- Epicanthal folds	+	-	+	+	+	+	+	+	+	+
- Palpebral ptosis	+	+	+	+	+	-	+	-	-	+
- Flat nasal bridge	+	-	+	+	+	+	+	-	+	+
- Broad nasal root	-	-	+	-	+	+	+	-	+	+
- Prominent philtrum	+	-	+	-	+	+	+	+	-	+
- Thick lips/macrostomia	-	-	-	-	+	-	+	-	-	+
- Low-set ears	+	+	+	+	+	+	+	+	+	+
- Thickened helix	-	-	+	+	+	+	+	-	+	+
- Large, thick ear lobe	-	-	+	+	+	+	+	-	+	+

Case	NS1	NS2	NS3	NS4	NS5	NS6	NS7	NS8	NS9	NS10
- Micrognathia	-	-	+		-	+	-	+	-	+

Supp. Table S3. Clinical features of *SOS1* mutation-positive subjects (continued)

Case	NS11	NS12	NS13	NS14	NS15	NS16	NS17	NS18	NS19	NS20
Code	BO-1651	BO-0596	BO-2635	BO-4338	HD1256	HD290	05-0175	07-0934	HD1284	HD696
Sex	M	M	F	M	F	M	M	F	F	F
Age at evaluation	4 y, 11 m	16 y, 7 m	26 y	1 y, 3 m	9 y, 7 m	22 y	13 y	10 y	6 y, 6 m	9 y
Amino acid change	W432R	E433K	R552G	E846K	R552S	K170E	L490R	R552G	I437T	R552G
Polyhydramnios	-	+	-	+	+	+	-	-	-	-
Fetal macrosomia	-	-	-	-	+		-	-	-	-
Neonatal/infantile growth failure	-	+	+	+	+	-	+	+	+	-
Poor sucking	-	-	+	+	-	-	-	-	N.R.	-
Poor swallowing	-	-	-	+		-	+	-	N.R.	-
Apneas	-	-	-	-	-	-	-	-	-	-
Short stature (<3rd centile)	-	+	-	+	+	-	-	-	+	-
Height/length (cm)	114	161	154	72	123	172	138.5	107	93	132.5
Age	4 y, 11 m	16 y, 7 m	26 y	1 y, 3 m	9 y, 7 m	22 y	11 y	6 y, 8 m	4 y, 6 m	8 y, 6 m
Centile	90th-97th	2nd	10th	2nd	< 3rd	25th	25th	3rd	<2nd	50th – 75th
Delayed bone age	-	+	+		+		-		+	-
GH deficiency	-	+	-		-	-	-			-
Craniofacial anomalies	+	+	+	+	+	+	+	+	+	+
- Macrocephaly	+	+	+	+	-	+	+	+	+	+
- Scaphocephaly	-	-	-	-	-	-	-	-	-	-
- High forehead	+	+	+	+	+	-	+	+	+	+
- Bitemporal narrowing	-	-	-	-	+	-	+	+	+	-
- Prominent supraorbital ridge	-	+	+	+	+	+	-	-	+	-
- Downslanting palpebral fissures	+	+	+	+	-	+	+	+	+	+
- Hypertelorism	+	+	+	+	-	+	+	+	+	+
- Epicanthal folds	-	+	+	-	+	-	+	-	+	+
- Palpebral ptosis	+	+	+	+	+	+	+	+	-	-
- Flat nasal bridge	-	-	-	-	+	-	+	+	+	-
- Broad nasal root	-	+	-	-	+	-	-	+	+	+
- Prominent philtrum	-	+	-	-	+	-	+	+	-	+

**Case**	**NS11**	**NS12**	**NS13**	**NS14**	**NS15**	**NS16**	**NS17**	**NS18**	**NS19**	**NS20**
- Thick lips/macrostomia	-	-	+	+	-	-	+		+	+
- Low-set ears	+	+	+	+	-	+	+	+	+	+
- Thickened helix	-	-	-	-	+	+	-	+	+	+
- Large, thick ear lobe	-	+	+	+	-	-	+	+	+	+
- Micrognathia	+	+	-	-	+	-	-	+	+	-

Supp. Table S3. Clinical features of *SOS1* mutation-positive subjects (continued)

Case	NS21	NS22	NS23	NS24	NS25	NS26	NS27	NS28	NS29	NS30
Code	N1183	N118 3_father	08-0045	N1157	N1212	N1185	HD227	HD215_father	HD215	HD715
Sex	M	M	M	M	M	F	F	M	F	M
Age at evaluation	12 y, 8 m	40 y	3 y	28 y	11 y, 7 m	15 y, 3 m	7 y, 4 m	37 y	3 y	1 y, 5 m
Amino acid change	P894R	P894R	G434K	M269T	R552M	C441Y	S548R	L550P	L550P	R552G
Polyhydramnios	+	N.R.	-	N.R.	-	+	-	-	+	+
Fetal macrosomia	+	-	-	-	-	+	+	-	-	+
Neonatal/infantile growth failure	-	-	-	N.R.	+	+	+	-	+	+
Poor sucking	-	-	-	-	-	+	-	-	+	+
Poor swallowing	-	-	-	-	-	+	-	-	+	+
Apneas	-	-	-	-	-	+	-	-	-	+
Short stature (<3rd centile)	+	+	-	-	+	+	-	-	-	+
Height/length (cm)	137cm	160cm	96cm	170cm	131.8cm	147 cm	118	171	93	79
Age	12 y, 8 m	40 y	3 y	28 y	11 y, 7 m	15 y, 3 m	7 y, 4 m	37 y	3 y	1 y, 5 m
Centile	0.4th – 2nd	0.4th – 2nd	75th	9th – 25th	0.4th – 2nd	< 3rd	25th	25th	50th	25th
Delayed bone age	+		-	-	+	+	+		+	+
GH deficiency	-		-	-	-	-	-		-	-
Craniofacial anomalies	+	+	+	+	+	+	+	+	+	+
- Macrocephaly	+	+	-	+	-	-	-	+	+	+
- Scaphocephaly	-	-	-	-	-	-	-	-	-	-
- High forehead	+	-	-	+	+	-	+	+	+	-
- Bitemporal narrowing	-	-	-	+	+	-	+	+	+	-
- Prominent supraorbital ridge	-	-	+	+	-	+	+	+	+	-
- Downslanting palpebral fissures	-	+	-	+	+	+	+	+	+	+
- Hypertelorism	+	+	+	+	-	+	+	+	+	+
- Epicanthal folds	-	-	-	-	+	+	+	-	-	-

Case	NS21	NS22	NS23	NS24	NS25	NS26	NS27	NS28	NS29	NS30
- Palpebral ptosis	+	+	+	+	+ (#)	+	+	+	+	+
- Flat nasal bridge	-	-	-	+	+	+	+	-	+	+
- Broad nasal root	-	-	-	-	-	-	-	-	-	-
- Prominent philtrum	-	+	+	-	+	+	-	-	+	+
- Thick lips/macrostomia	+	+	+	+	-	+	+	-	+	+
- Low-set ears	+	+	+	+	+	+	+	+	+	+
- Thickened helix	+	+	+	+	+	+	+	+	+	+
- Large, thick ear lobe	+	-	-	-	+	+	-	-	+	+
- Micrognathia	-	-	-	-	+	+	-	-	-	-

Supp. Table S3. Clinical features of *SOS1* mutation-positive subjects (continued)

Case	NS31	NS32	NS33	NS34	NS35	NS36	NS37	NS38	NS39	Total
Code	HD303	04-0400	HD099	S1073	S1089	HD1090	S1108	S1121	S1135	
Sex	M	M	F	M	M	F	M	F	F	25 M, 14F
Age at evaluation	3 y, 5 m	8 y, 2 m	5 y, 0 m	18 y	34 y	13 y, 7 m	17 y	6 y	3 y	8 y, 10 m^{+}
Amino acid change	R552G	R552G	W729L	R552G	R552G	R552S	R552S	R552K	I733F	
Polyhydramnios	-	+	+	-	+	+	-	-	+	18/37 (49%)
Fetal macrosomia	+	+	-	+	+	-	+	+	-	14/38 (37%)
Neonatal/infantile growth failure	+	-	+	-	-	-	-	-	-	17/35 (49%)
Poor sucking	+	-	+	-	-	-	-	-	-	9/38 (24%)
Poor swallowing	+	-	+	-	-	-	-	-	-	8/37 (22%)
Apneas	-	-	+	-	+	-	-	-	-	5/37 (12%)
Short stature (<3rd centile)	-	-	-	-	-	-	-	-	-	11/39 (28%)
Height/length (cm)	100	115	102	73.5	165	154		118		
Age	3 y, 5 m	8 y, 2 m	5 y, 0 m	20 y	34 y, 7 m	13 y, 7 m		8 y, 1 m		
Centile	75th	<3rd	10th	25th-50th	5th	25th	5th	5th	10th	
Delayed bone age	+	+	-	-	-	+	-	-	-	15/26 (58%)
GH deficiency	-	-	-	-	-	-	-	-	-	3/26 (12%)
Craniofacial anomalies	+	+	+	+	+	+	+	+	+	39/39 (100%)
- Macrocephaly	+	+	+	-	+	-	+	-	+	24/39 (61%)
- Scaphocephaly	-	-	-	-	-	-	-	-	-	0/39 (0%)
- High forehead	-	-	+	-	-	-	-	-	+	25/39 (64%)
- Bitemporal narrowing	-	-	+	-	-	-	-	-	-	17/39 (44%)

Case	NS31	NS32	NS33	NS34	NS35	NS36	NS37	NS38	NS39	Total
- Prominent supraorbital ridge	-	-	+	-	+	-	+	+	+	22/39 (56%)
- Downslanting palpebral fissures	+	+	+	+	+	+	+	+	+	35/39 (90%)
- Hypertelorism	+	+	+	+	+	+	+	+	+	34/39 (87%)
- Epicanthal folds	+	-	+	+	+	+	+	+	+	26/39 (67%)
- Palpebral ptosis	+	+	+	+	+	+	+	+	+	34/39 (87%)
- Flat nasal bridge	+	+	+	+	+	+	+	+	+	27/39 (69%)
- Broad nasal root	-	-	-	-	+		-	+	+	14/38 (37%)
- Prominent philtrum	-	+	-	-	+	-	+	-	+	22/39 (56%)
- Thick lips/macrostomia	+	+	+	+	+	+	+	+	+	25/38 (66%)
- Low-set ears	+	+	+	+	+	+	+	+	+	38/39 (97%)
- Thickened helix	+	+	+	+	+	+	-	+	+	30/39 (77%)
- Large, thick ear lobe	-	-	+	+	+	+	+	+	+	25/38 (66%)
- Micrognathia	-	-	-	-	-	-	+	-	-	12/38 (32%)

Supp. Table S3. Clinical features of *SOS1* mutation-positive subjects (continued)

**Case**	**NS1**	**NS2**	**NS3**	**NS4**	**NS5**	**NS6**	**NS7**	**NS8**	**NS9**	**NS10**
Congenital heart defects	+	+	+	+	+	+	+	-	+	+
- Pulmonic stenosis (PS)	+	+	+	+	-	+	+	-	+	+
- Pulmonary valve dysplasia	-	-	-	-	-	-	-	-	-	-
- Mitralic valve dysplasia	-	-	-	-	-	-	-	-	-	-
- Supravalvular PS	-	-	-	-	-	-	-	-	-	-
- Atrial septal defect	-	-	+	-	-	-	-	-	-	-
- Hypertrophic cardiomyopathy	-	-	-	-	-	-	-	-	-	-
- Atrioventricular canal	-	-	-	-	-	-	-	-	-	-
- Tetralogy of Fallot	-	-	-	-	-	-	-	-	-	-
- Ventricular septal defect	-	-	-	-	+	-	-	-	-	+
- others	-	-	-	-	-	-	-	+ (Ar)	-	-
Skin features	-	-	+	-	-	-	+	-	+	+
- Dark skin	-	-	-	-	-	-	-	-	+	+
- palmo/plantar crease	-	-	+	-	-	-	-	-	-	-
- Pruritus	-	-	-	-	-	-	+	-	-	-
- others	-	-	-	-					-	
Ectodermal features	+	-	+	-	+	+	+	+	+	+

Case	NS1	NS2	NS3	NS4	NS5	NS6	NS7	NS8	NS9	NS10
- Sparse/absent scalp hair	-	-	-	-	-	-	+	-	-	-
- Thin hair	-	-	-	-	-	+	+	-	-	-
- Curly hair	+	-	-	-	+	-	-	+	-	+
- Sparse eyebrows	+	-	+	-	+	+	+	+	+	+
- Keratosis pilaris faciei	+	-	-	-	-	-	-	-	-	-
- Thin dystrophic nails	-	-	-	-	-	-	-	-	-	-
Musculoskeletal abnormalities	+	-	+	+	+	+	+	+	+	+
- Short webbed neck	+	-	+	+	+	+	+	+	+	+
- Cubitus valgus	-	-	-	-	+	+	+	+	+	+
- Hyperextensible joints	-	-	-	-	-	-	-	-	-	+
- Pectus anomalies	-	-	+	+	+	+	+	+	+	+
Undescended testes	+	-		+	+	+	+	+	+	+
CNS involvement	-	+	-	+	-	-	-	-	-	+
- Mental retardation	-	+ (*)	-	-	-	-	-	-	-	+ (**)
- Hyperactivity	-	-	-	-	-	-	-	-	-	-
- CNS abnormalities	-	-	-	-	-	-	-	-	-	+ (ArCy)
- Seizures/EEG abnormalities	-	-	-	-	-	-	-	-	-	+

Supp. Table S3. Clinical features of *SOS1* mutation-positive subjects (continued)

Case	NS11	NS12	NS13	NS14	NS15	NS16	NS17	NS18	NS19	NS20
Congenital heart defects	+	+	+	+	+	+	+	+	+	+
- Pulmonic stenosis (PS)	-	-	+	+	+	+	+	+	+	+
- Pulmonary valve dysplasia	-	-	-	-	+	-	-	-	+	-
- Mitralic valve dysplasia	-	-	-	-	+	-	-	-	-	-
- Supravalvular PS	-	-	-	-	-	-	-	-	-	-
- Atrial septal defect	-	-	+	-	-	-	-	+	+	+
- Hypertrophic cardiomyopathy	-	-	-	-	-	-	-	-	+ (LV)	-
- Atrioventricular canal	-	-	-	-	-	-	-	-	-	-
- Tetralogy of Fallot	-	-	-	-	-	-	-	-	-	-
- Ventricular septal defect	-	+	-	-	-	-	-	-	+	-
- others	+ (MVP, MVI)	-	-	-	-	-	-	-	-	-
Skin features	-	-	+	+	+	+	-	+	-	-
- Dark skin	-	-	+	-	-	-	-	-	-	-

Case	NS11	NS12	NS13	NS14	NS15	NS16	NS17	NS18	NS19	NS20
- palmo/plantar crease	-	-	+	+	-	-	-	+		-
- Pruritus		-	-	+	-	-	-		-	-
- others	-		+ (SK)		+ (DrSk)	+ (K)				
Ectodermal features	+	+	+	+	+	+	+	+	+	+
- Sparse/absent scalp hair	+	-	+	+	+	-	-	-	-	-
- Thin hair	+	+	+	+	+	-	-	+	+	+
- Curly hair	-	+	+	+	+	+	-	-	-	+
- Sparse eyebrows	+	+	+	+	+	+	+	+	+	+
- Keratosis pilaris faciei	-	-	+	-	-	+	-	-	-	-
- Thin dystrophic nails	-	-	-	-	+	-	-	-		-
Musculoskeletal abnormalities	+	-	+	+	+	+	+	+	+	+
- Short webbed neck	-	-	+	+	+	+	-	+	+	+
- Cubitus valgus	-	-	-	-	-	-	-			-
- Hyperextensible joints	-	-	+	+	+	-		+		-
- Pectus anomalies	+	-	+	+	-	+	+ (PE)	+ (PC)	+ (PE)	-
Undescended testes	-	-		-		+	-			
CNS involvement	-	-	-	-	-	-	-	+	-	-
- Mental retardation	-	-	-	-	-	-	- (***)	+	- (****)	- (^)
- Hyperactivity	-	-	-	-	-	-	-			-
- CNS abnormalities	-	-	-	-		-	-	-	-	-
Seizures/EEG abnormalities	-	-	-	-	-	-	-	-	-	-

Supp. Table S3. Clinical features of *SOS1* mutation-positive subjects (continued)

Case	NS21	NS22	NS23	NS24	NS25	NS26	NS27	NS28	NS29	NS30
Congenital heart defects	-	-	+	+	+	+	+	-	+	+
- Pulmonic stenosis (PS)	-	-	+	+	-	+	-	-	+	+
- Pulmonary valve dysplasia	-	-	+	-	-	+	-	-	+	-
- Mitralic valve dysplasia	+	-	-	-	+	-	-	-	-	+
- Supravalvular PS	-	-	-	-	-	-	-	-	+	-
- Atrial septal defect	-	-	+	-	-	-	+	-	-	-
- Hypertrophic cardiomyopathy	-	-	-	-	-	-	-	-	-	-
- Atrioventricular canal	-	-	-	-	-	-	-	-	-	-
- Tetralogy of Fallot	-	-	-	-	-	-	-	-	-	-

Case	NS21	NS22	NS23	NS24	NS25	NS26	NS27	NS28	NS29	NS30
- Ventricular septal defect	-	-	-	-	-	-	-	-	-	-
- others	-	-	-	-	AVD	MVP,AVDRCoTA	-	-	-	-
Skin features	+	+	+	-	-	-	+	+	+	+
- Dark skin	+	+	+	-	-	-	+	+	+	+
- Palmo/plantar crease	-	-	-	-	-	-	-	-	-	+
- Pruritus	+	-	-	-	-	-	-	-	-	+
- others	-	-	-		-					+ (CA)
Ectodermal features	+	+	+	+	-	+	+	+	+	+
- Sparse/absent scalp hair	-	-	-	-	-	-	-	-	-	-
- Thin hair	-	-	-	-	-	-	-	-	-	-
- Curly hair	+	+	+	+	-	+	+	+	+	+
- Sparse eyebrows	+	-	+	-	-	+	+	-	+	+
- Keratosis pilaris faciei	+	-	+	-	-	+	-	-	-	-
- Thin dystrophic nails	-	-	-	-	-	-	-	+	-	-
Musculoskeletal abnormalities	+	+	+	+	+	+	+	+	+	+
- Short/webbed neck	-	+	-	-	+	+	+	-	+	+
- Cubitus valgus	-	-	-	-	-	+	-	+	+	-
- Hyperextensible joints	+	+	+	-	-	+	+	+	+	+
- Pectus anomalies	+	-	-	+	+	+	+	+	+	+
Undescended testes	+	-	+	+	+			+		+
CNS involvement	-	-	-		-	+	-	-	-	-
- Mental retardation	-	-	-	-	-	+ (*****)	-	-	-	-
- Hyperactivity	-	-	-	-	-	+	-	-	-	-
- CNS abnormalities	-	-	-	-	-	-	-		-	-
- Seizures/EEG abnormalities	-	-	-	-	-	-	-	-	-	-

Supp. Table S3. Clinical features of *SOS1* mutation-positive subjects (continued)

Case	NS31	NS32	NS33	NS34	NS35	NS36	NS37	NS38	NS39	Total
Congenital heart defects	+	+	+	+	+	+	+	+	+	35/39 (90%)
- Pulmonic stenosis (PS)	-	+	+	+	-	+	+	+	-	27/39 (69%)
- Pulmonary valve dysplasia	+	+	+	-	-	+	+	+	-	11/39 (28%)
- Mitralic valve dysplasia	+	-	-	-	-	-	-	-	+	6/39 (15%)
- Supravalvular PS	-	+	+	-	-	+	-	-	-	4/39 (10%)

**Case**	**NS31**	**NS32**	**NS33**	**NS34**	**NS35**	**NS36**	**NS37**	**NS38**	**NS39**	**Total**
- Atrial septal defect	-	+	+	+	-	-	-	-	-	10/39 (26%)
- Hypertrophic cardiomyopathy	+	-	-	-	+	-	-	-	-	3/39 (8%)
- Atrioventricular canal	-	-	-	-	-	-	-	-	-	0/39 (0%)
- Tetralogy of Fallot	-	-	-	-	-	-	-	-	-	0/39 (0%)
- Ventricular septal defect	-	-	-	-	-	+	-	-	-	5/39 (13%)
- others	-	-	-	-	-	-	-	-	-	
Skin features	+	+	+	-	+	+	+	-	-	22/39 (57%)
- Dark skin	+	+	+	-	+	+	+	+	+	18/39 (46%)
- palmo/plantar crease	-	-	-	-	+	-	-	+	-	7/38 (18%)
- Pruritus	+	+	+	-	-		-	+	-	8/36 (22%)
- others							-		+ (CA)	
Ectodermal features	+	+	+	+	-	+	+	+	+	35/39 (90%)
- Sparse/absent scalp hair	-	-	-	-	-	-	-	+	+	7/39 (18%)
- Thin hair	-	-	-	-	-	-	-	+	+	12/39 (31%)
- Curly hair	+	+	+	+	-	+	+	+	+	27/39 (69%)
- Sparse eyebrows	-	+	+	-	-	+	+	+	+	30/39 (77%)
- Keratosis pilaris faciei	+	+	+	-	+	+	+	+	-	13/39 (33%)
- Thin dystrophic nails	-	-	-	-	+	-	-	+	-	4/38 (10%)
Musculoskeletal abnormalities	+	+	+	+	+	+	+	+	+	37/39 (95%)
- Short webbed neck	+	+	+	+	+	+	+	+	+	31/39 (79%)
- Cubitus valgus	-	-	+	+	+	+	+	+	+	16/37 (43%)
- Hyperextensible joints	+	+	+	-	-	-	-	+	-	17/37 (46%)
- Pectus anomalies	+	+	+	+	+	+	+	+	+	32/39 (82%)
Undescended testes	-	+		+	+		-			18/26 (69%)
CNS involvement	-	-	-	-	-	-	-	-	-	5/38 (13%)
- Mental retardation	-	-	-	-	-	-	-	-	-	4/39 (13%)
- Hyperactivity	-	-	-	-	-	-	-	-	-	1/37 (3%)
- CNS abnormalities	-	-	-	-	AC	-	-	-	-	2/37 (5%)
- Seizures/EEG abnormalities	-	-	-	-	-	-	-	-	-	1/39 (3%)

Supp. Table S3. Clinical features of *SOS1* mutation-positive subjects (continued)

Case	NS1	NS2	NS3	NS4	NS5	NS6	NS7	NS8	NS9	NS10
Ocular abnormalities	-	-	+ (Sb)	+ (Ast)	-	+ (Sb)	-	+ (My)	-	+
Gastrointestinal abnormalities	-	-	+ (GER)	-	-	-	-		-	-
Renal abnormalities	-	-	-	-	-	-	-		-	-
Coagulation defects	-	-	-	-	+ (VWD)	-	-		-	-
Mandibular giant cell lesions	-	-	-	-	-	-	-	-	-	-
Other tumors	-	-	-	-	-	-	-	-	-	-
Others	-	-	-	-	-	-	-	IH	-	-

Supp. Table S3. Clinical features of *SOS1* mutation-positive subjects (continued)

Case	NS11	NS12	NS13	NS14	NS15	NS16	NS17	NS18	NS19	NS20
Ocular abnormalities	-	-	+ (lC, My)	-	-	-	-	+ (My, Ast,Sb)	-	-
Gastrointestinal abnormalities	-	-	-	-	-	-	-	-	-	+ (GER solv.)
Renal abnormalities	-	+ (BH)	+ (lN)	-	-	-	+ (ERP)	-	-	-
Coagulation defects			-			+	-	+ (CPD)	+(##)	-
Mandibular giant cell lesions	-	-	-	-	-	-	-	-	-	+
Other tumors	-	-	-	-	-	-	-	-	-	-
Others	-	-	V, CoHipD	luDy, AbU	SFF	LTT	-	-	-	-

Supp. Table S3. Clinical features of *SOS1* mutation-positive subjects (continued)

Case	NS21	NS22	NS23	NS24	NS25	NS26	NS27	NS28	NS29	NS30
Ocular abnormalities	-	-	-	-	-	+	-	-	-	-
Gastrointestinal abnormalities	-	-	-	-	-	-	-	-	-	-
Renal abnormalities	-		-	-	-	-	+	-	-	-
Coagulation defects	-	-	-	-	-	-	+	-	-	-
Mandibular giant cell lesions	+	-	-	-	-	-	-	-	-	-
Other tumors	abRMS, cGl, GCC	-	-	-	-	-	-	-	-	-
Other	N, L, HypThy	-	HTGum	-	-	-	-	-	-	-

Supp. Table S3. Clinical features of *SOS1* mutation-positive subjects (continued)

Case	NS31	NS32	NS33	NS34	NS35	NS36	NS37	NS38	NS39	Total
Ocular abnormalities	-	-	+ (Sb)	+	+	-	+	+	+	13/39 (33%)
Gastrointestinal abnormalities	-	-	-	-	+	-	-	-	-	3/38 (8%)
Renal abnormalities	-	-	-	-	-	-	-	-	-	4/38 (10%)
Coagulation defects	-	+	-	-	+	+	-	+	-	9/34 (26%)
Mandibular giant cell lesion	-	-	-	-	-	+	-	-	-	3/39 (8%)
Other tumors	-	-	-	-	-	-	-	-	-	1/39 (3%)
Others	-	-	-	-	-	ArS	-	-	-	

abRMS= abdominal rhabdomyosarcoma; AbU= absence of uvula; AC= Arnold-Chiari malformation; Ar= arhythmia; ArCy= arachnoid cyst; ArS= articular synovitis; Ast= astigmatism; AVD= aortic valve dysplasia; BH= bilateral hydronephrosis; CoHipD= congenital hip dysplasia; CA= Cavernous angiomata; cGl= cerebral glioma; CPD= coagulation-platelet disfunction; DrSk= dry skin; DUT= Double urinary tract; ERP= enlarged renal pelvis; F= female; GCC= granular cell tumors; GER= gastroesophageal reflux ; HypoGly= Hypoglycemia; HypThy= hypothyroidism; HTGum= Hypertrophic gums; IH= inguinal hernia; K= keloid; L= lentigines; lC= left cataract; lN= left nephrolithiasis; luDy= 1st lumbar and 4th cervical vertebral dysmorhism; LV= left ventricle; LTT= large thumbs and toes; M= male; MVI= mitral valve insufficiancy; MVP= mitral valve prolapse; My= Myopia; N= nevi; PC= pectus carenatum; PE= pectus excavatum; PP= pes planovalgus; RCoTA= right cor triatriatum ; s= severe; Sb= strabismus; solv.= solved; SK= skin keloids; SFF= severe flat feet; TI= tricuspid insufficiency; V= valgism; VWD= von willebrand disease.

[+]Median value (range: 5 months to 40 years), [#]More prominent on the left eye; [##]Coagulation factors VII, IX, and XI deficiency, ^ First steps at 16-17 months of age; *The subject had hypoxia as a complication of the heart surgery; ** Developmental delay was documented, with unsupported walking reached at 18-20 months and first spoken words at 30 months. Cognitive development was impaired, with requirement of a full time special education setting. Poor fine motor skill was also reported; ***Speech delay; ****Wechsler Preschool and Primary Scale of Intelligence Total Intellective Quotient = 81; at 4 years, according to the Griffith's scale, the General Quotient was border-line to the inferior normal limits, with impairment of linguistic skills and oculo-manual coordination; *****Cognitive deficits were secondary to surgery complication during the neonatal period (IQ score= 44, WISC-R scale).
